# Targeting the MDM2‐MDM4 interaction interface reveals an otherwise therapeutically active wild‐type p53 in colorectal cancer

**DOI:** 10.1002/1878-0261.70006

**Published:** 2025-02-28

**Authors:** Sonia Valentini, Giada Mele, Marika Attili, Maria Rita Assenza, Fulvio Saccoccia, Francesca Sardina, Cinzia Rinaldo, Roberto Massari, Nicola Tirelli, Alfredo Pontecorvi, Fabiola Moretti

**Affiliations:** ^1^ Institute of Biochemistry and Cell Biology National Research Council of Italy Monterotondo Italy; ^2^ PhD Course in Sciences of Nutrition, Aging, Metabolism and Gender Pathologies Catholic University of Roma Italy; ^3^ Institute of Molecular Biology and Pathology National Research Council of Italy Roma Italy; ^4^ Laboratory of Polymers and Biomaterials Fondazione Istituto Italiano di Tecnologia Genova Italy; ^5^ Department of Medicine and Translational Surgery Catholic University of Roma Italy; ^6^ Present address: Department of Medicine and Surgery “Kore” University of Enna Italy

**Keywords:** colorectal cancer, MDM2/MDM4 heterodimer, Nutlin‐3a, p53, therapeutic peptide

## Abstract

Targeting the heterodimer MDM2/MDM4 is a novel and effective route for the reactivation of wild‐type p53 in human tumors with reduced toxicity in nontransformed cells. To improve the therapeutic potential of peptides that interfere with MDM4 binding to MDM2, we demonstrated the tumor‐suppressive activity of a short peptide (Pep3S), which is composed of the last five amino acids of the MDM4 protein. Compared to longer peptides (previously identified), Pep3S binds MDM2 with high affinity, increases p53‐dependent cell death in 2D and 3D colorectal cancer models, and is more efficacious in suppressing xenograft tumor growth. Furthermore, its encapsulation in poly (lactic‐co‐glycolic acid) (PLGA) nanoparticles potentiated and prolonged its activity. A p53‐specific target gene array revealed an uncommon p53 signature, with Pep3S leading to p53‐mediated repression of a subset of p53 targets. Comparative analysis indicated that this repression is driven by p53‐mediated activation of miR‐34a, which is functional in Pep3S‐induced cell death. Of note, unlike other p53‐reactivating molecules, Pep3S led to significant downregulation of the cell cycle inhibitor CDKN1A/p21, one of the best‐characterized p53‐targets. Genetic manipulation of MDM4 demonstrated the requirement of the dissociated protein for p21 downregulation, whereas the miR‐34a signature was not altered. At odds with Nutlin‐3a, the proliferation status of nontumor muscle and lymphoblastoid cells was not altered by Pep3S. These data indicate that targeting the MDM2/MDM4 interaction region provides a different route for wild‐type p53 reactivation in human tumors, potentially reducing toxicity to proliferating nontumor tissue. The development of a PLGA/Pep3S formulation represents a promising approach for therapeutic purposes.

## Graphical abstract

This study investigates an alternative approach to reactivating the oncosuppressor p53 in cancer. A short peptide targeting the association of the two p53 inhibitors, MDM2 and MDM4, induces an otherwise therapeutically active p53 with unique features that promote cell death and potentially reduce toxicity towards proliferating nontumor cells. Peptide conjugation with nanoparticles provides an additional valuable tool for peptide‐based therapy.

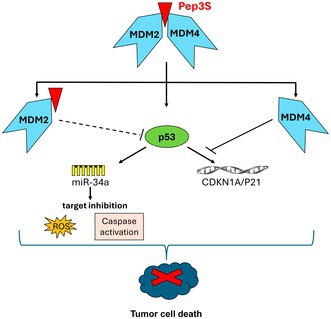

**Abbreviations**
ADMET: absorption, distribution, metabolism, and excretion–toxicity
BF: bright field
CRC: colorectal cancer
DCF: dichlorofluorescein
DEGs: differentially expressed genes
DLS: dynamic light scattering
DOX: doxycycline
FITC: fluorescein isothiocyanate
GEPIA2: gene expression profiling interactive analysis
H2DCFDA: 2′,7′‐dichlorofluorescein diacetate
LCLs: immortalized lymphoblastoid cells
MDM2: mouse double minute 2 homolog
MDM4: double minute 4 protein
MSI‐H: high microsatellite instability
MSI‐L: low microsatellite instability
MSS: microsatellite stability
NPs: nanoparticles
NTA: nanoparticle tracking analysis
PDI: polydispersity
PI: propidium iodide
PLGA: poly(lactic‐co‐glycolic acid)
RING: really interesting new gene
ROI P/s: region of interest photon/s
RT‐qPCR: quantitative reverse transcription polymerase chain reaction
sc: subcutaneous
sh: short hairpin
TGCA: Cancer Genome Atlas Program
wt: wild‐type

## 1 Introduction

The oncosuppressor p53 has long been an appealing target in the cancer field [1]. In cancers expressing wild‐type p53, the assumption that its function is impaired has led to the development of a comprehensive list of re‐activators [2]. Since the discovery of MDM2, the best‐characterized p53 negative regulator, many molecules have been developed with therapeutic potential [3]. The first class is represented by cis‐imidazoline compounds, Nutlins, small molecules that bind MDM2 and inhibit its binding to p53 [4]. Inhibition of MDM2‐p53 binding leads to the loss of p53 degradation by the ubiquitin ligase activity of MDM2 and the release of its transcriptional function. Following Nutlins, many derivatives and other molecules have been tested in clinical trials. Unfortunately, their clinical use has not been reached [3, 5]. The discovery of a second inhibitor of p53, MDM4 (also MDMX), partially explained this failure [6, 7] and reshaped strategies for reactivating p53 function in human tumors. MDM2 inhibitors were retested on MDM4, and novel dual inhibitors were developed [8]. Some of these dual inhibitors have progressed through clinical phases but have not yet reached the patient's bed.

One adverse effect that impairs the advancement of some p53‐reactivating molecules is their toxicity to nonmalignant cells [2, 5]. Adverse effects mainly include bone marrow cytopenia and gastrointestinal disorders [9, 10]. The proliferative status of these tissues is a potential cause of their failure since the activation of p53 induces CDKN1A/p21/WAF1, a crucial mediator of growth arrest [9]. In fact, Nutlin‐3a and its derivatives have been used in combination therapy to treat polycythemia [10].

Following MDM4 discovery, the heterodimer composed of MDM2 and MDM4 appeared to be a more efficient inhibitor of p53 than single proteins [11, 12]. The binding between MDM2 and MDM4 potentiates the degradative activity of MDM2 and is required for efficient inhibition of p53 transcriptional activity by MDM4 [13]. In addition, single proteins appear to carry out different activities towards p53 [12]. Particularly, MDM4 inhibitory activity is less intense than that of MDM2 [14] and primarily controls p53‐mediated growth arrest in some tissues [15, 16]. Moreover, under lethal DNA damage it positively affects the active conformation of p53 and apoptotic response [17, 18, 19].

Consequently, the hypothesis was proposed to target heterodimer activity and release single proteins. Unfortunately, the MDM2/MDM4 interaction region is difficult to target by small molecules because it encompasses a relatively flat β‐sheet surface. In 2015, the development of a peptide impairing the MDM4/MDM2 association and increasing the oncosuppressive activity of p53 supported the heterodimerization surface as a druggable region [20]. Interestingly, this peptide did not heavily increase the levels of p53 and was ineffective in nontransformed cells, supporting the hypothesis of reduced toxicity in healthy tissues [20]. Despite this initial report, few studies have been developed to target the MDM2/MDM4 interaction region [21].

Based on molecular docking studies, we recently designed a novel short peptide encompassing the last five C‐terminal amino acids of human MDM4, Pep3S. The dissociation constant of this short peptide from human MDM2 was approximately half that of the previous long peptide (Pep3), indicating higher affinity.

In this work, we tested the biological activity of this novel short peptide compared to the longer one and exploited its efficiency by conjugation with nanoparticles. Interestingly, this peptide showed activation of a different p53‐mediated signature compared to Nutlin‐3a, supporting the MDM2/MDM4 interaction surface as a different route for wt‐p53 reactivation in human tumors.

## 2 Materials and methods

### 2.1 Peptides

Lyophilized Pep3S (KVFIA) and the control peptide Scramble3S, constituted by the same amino acids in a different sequence (VAIKF), were obtained from the Department of Pharmaceutical Sciences, University of Perugia. Lyophilized Pep3 (Ac‐KEIQLVIKVFIA‐NH2), Scramble3 (Ac‐VIFVIKAKEIQL‐NH2), and Pep3S‐FITC (FITC‐KVFIA‐NH2) were synthesized from SynPeptide (Shanghai, China). The peptide's purity level was ≥ 96%. All peptides were dissolved in 100% DMSO at a concentration of 10 mm. For *in vitro* studies, they were first solubilized in sterile Milli‐Q grade H_2_O at the concentration of 0.5 mm and then premixed with growth media at 37 °C for 15 min to a final concentration of 10 μm.

### 2.2 Data set analysis

Gene expression data were analyzed and visualized by GEPIA2 (Gene expression profiling Interactive analysis) [22], using RNA sequencing datasets from TCGA. Transcript levels are expressed as lg_2_(TPM + 1). The applied lg_2_ fold change was > 0.58.

### 2.3 Cell culture and treatment

Human colorectal carcinoma cells HCT116 wt‐p53 (RRID: CVCL_0291) were purchased from Sigma‐Aldrich (Darmstadt, Germany). HCT116 exon two knockout TP53 (hereafter p53^−/−^) were obtained from Vogelstein's Lab, LoVo (Johns Hopkins University, Baltimore, MD, USA) (RRID: CVCL_0399) from Vitiani's lab (Istituto Superiore di Sanità, Roma, Italy). HCT116 and LoVo cells were maintained in DMEM low glucose (Sigma‐Aldrich) supplemented with 10% FBS (Gibco™, Walthman, MA, USA), 1% penicillin–streptomycin (Sigma‐Aldrich), 1% l‐glutamine (Sigma‐Aldrich) at 37 °C, 5% CO_2_. Lymphoblastoid cells from healthy donors (LCLs) [23] were grown in RPMI‐1640 (Sigma‐Aldrich), supplemented with 15% FBS (Gibco™), 1% penicillin–streptomycin (Sigma‐Aldrich), 1% l‐glutamine (Sigma‐Aldrich). Primary human myoblasts (Lonza, Basel, Switzerland) were grown in SkGM‐2 medium (Lonza). Cell lines were maintained at 37 °C, 5% CO_2_.

Mycoplasma‐free conditions were routinely tested using a MycoAlert kit (Lonza), and all experiments were performed with mycoplasma‐free cells. Cell lines have been authenticated within the past 3 years using the Eurofins Genomics CLA service.

Treatments (0.1% DMSO, 10 μm all peptides, 20 μm cisplatin, 10 μm Nutlin‐3a (Sigma‐Aldrich)) were administered every other day. For spheroids formation, 2.5 × 10^3^ cells per well were resuspended in 20 μL of complete medium supplemented with 1 mg·mL^−1^ methylcellulose (Sigma‐Aldrich). The cells were seeded in ultra‐low attachment round‐bottom 96‐well plates (Corning, Glendale, CA, USA or IWAKI, Tokyo, Japan) and centrifuged at 46 ***g***. for 5 min. The next day, 80 μL of complete medium was added, and in approximately 3 days, spheroids were formed.

### 2.4 Gene silencing

ShMDM4 and shLacZ (as control) HCT116 (wt‐p53 and p53^−/−^) cells were obtained by transfection of doxycycline‐inducible silencing plasmids using jetPRIME transfection reagent (Polyplus, Illkirch‐Graffenstaden, France). Cells were maintained in DMEM low glucose (Sigma‐Aldrich) supplemented with 10% FBS (Gibco™) and 1% penicillin–streptomycin (Sigma‐Aldrich). After 24 h, the cells were selected with 1 mg·mL^−1^ puromycin (Sigma‐Aldrich). Silencing was induced with 1 μg·mL^−1^ doxycycline.

The activity of miR‐34a was inhibited by mirVana® miRNA inhibitor (Ambion, Thermo Fisher Scientific, Walthman, MA, USA). HCT116 were transiently transfected with hsa‐miR‐34a‐5p mirVana 25 pmol or control siRNA using Lipofectamine® RNAiMAX (Thermo Fisher Scientific).

### 2.5 Viability and apoptosis analyses

For 2D cell culture, 2 × 10^3^ cells per well of LoVo and HCT116 were seeded into 96‐well white plates (Corning) in complete medium. For 3D cell culture, LoVo and HCT116 spheroids were transferred into a 96‐well white plate (Costar) after formation and then treated as 2D cells.

Cell viability was monitored every other day using PrestoBlue™ HS Cell Viability Reagent (Thermo Fisher Scientific). The fluorescence signal was read at 45′ (for 2D) or 1 h (for spheroids) using a spectrophotometer plate reader (Varioskan™ LUX; Thermo Fisher Scientific). Fluorescence excitation/emission: 570/610 nm.

For apoptosis detection, caspase activity was measured using the Apo‐ONE® Homogeneous Caspase‐3/7 Assay (Promega, Madison, WI, USA). Caspase inhibitor Z‐VAD (Promega) was added at a concentration of 10 μm. After reagent addition, the fluorescence signal was read at 30′ (for 2D) or 1 h (for 3D spheroids). Fluorescence excitation/emission: 485/530 nm. The results were normalized to the total protein using the Bradford Assay (Bio‐Rad, Hercules, CA, USA).

For reactive oxygen species analysis, HCT116 and LoVo cells were assayed 48 h after treatment. Cells were washed with 1× DPBS and incubated with 25 μm 2′,7′‐dichlorofluorescein diacetate (H2DCFDA, Sigma‐Merck) in serum‐free media at 37 °C for 30′. Cells were washed once in 1× DPBS and incubated for 3 h in complete medium supplemented with or without 100 mm H_2_O_2_. The fluorescence signal was monitored using a spectrophotometer plate reader (Varioskan™ LUX). Fluorescence excitation/emission: 495 nm/520 nm. The results were normalized to the number of cells per well using PrestoBlue™ HS Cell Viability Reagent (Thermo Fisher Scientific).

### 2.6 Long‐term cell growth assay

LoVo and HCT116 cells were plated in complete medium in 12‐well plates at a density of 10 × 10^3^ cells per well. Treatments were administered every other day until day 6. At the end of the experiment, the cells were harvested and plated in 35 mm dishes in complete medium. After 24 h, cells were washed twice in 1× DPBS, fixed in 100% methanol (Sigma‐Aldrich) for 10′, and stained with 0.5% crystal violet (Sigma‐Aldrich) solution in 20% methanol. Images were acquired using an Olympus BX41 Phase Contrast & Darkfield microscope, with a 10× objective, and analyzed using fiji‐imagej software.

### 2.7 Spheroids staining

For cell viability and cell death analyses, LoVo and HCT116 spheroids were incubated with 2 μm calcein (Sigma‐Aldrich), 4 μg·mL^−1^ propidium iodide (Sigma‐Aldrich), and 15 μg·mL^−1^ Hoechst 33342 for 1 h at 37 °C. The spheroids were washed twice with 1× DPBS and placed in 96‐well glass‐bottom plates (Corning). The images were acquired using an Olympus IX83 confocal microscope with a 10× objective and analyzed using fiji‐imagej software.

### 2.8 RNA preparation and RT‐PCR

Gene expression was evaluated 48 h after treatment using quantitative real‐time PCR (7900HT Fast; Applied Biosystems, Foster City, CA, USA). RNA extraction was performed using TRIzol® Reagent (Invitrogen, Waltham, MA, USA). RNA (500/1000 ng from cells or mice, respectively) was reverse transcribed with SMART MMLV Reverse Transcriptase (Takara, Shiga, Japan) and subjected to PCR amplification using the SensiMix SYBR Lox‐ROX Kit (Meridian Bioscience, Cincinnati, OH, USA). The primers used are listed in Table S1.

For human p53 signaling pathway analysis, the RT^2^ Profiler PCR Array 384 well ‘Human p53 signaling pathway’ was used, following the manufacturer's instructions (Qiagen, Venlo, The Netherlands). Differences of ≥ 1.3‐fold were used as exclusion criteria. The hsa‐miR‐34a expression was analyzed using the TaqMan™ MicroRNA Assay (Thermo). The results were normalized to those obtained using snRNA RNU6B. Common targets of p53 and miR‐34a were evaluated using miRGator v3.0, miRTarBase, and TargetScanHuman.

### 2.9 Western blot analysis

Cells were lysed using RIPA buffer (50 mm Tris/HCl pH 7.5, 150 mm NaCl, 1% NP‐40, 0.5% sodium deoxycholate, 0.1% SDS, 1 mm EDTA) supplemented with 1 mm PMSF (Sigma‐Aldrich), 1 mm Na_3_VO_4_ (Sigma‐Aldrich), 5 mm NaF (Sigma‐Aldrich), and 10 μL·mL^−1^ Protease inhibitor cocktail (Sigma‐Aldrich), incubated for 30′ on ice, and centrifuged 18 400 ***g*** for 30′ at 4 °C. The protein concentration was determined using the Bradford Assay (Bio‐Rad). Proteins were transferred onto polyvinylidene difluoride (PVDF) membranes (Millipore, Burlington, MA, USA). Membranes were developed using the enhanced chemiluminescence kit (ECL Amersham, GE Healthcare, Amersham, UK) by the chemiluminescence imaging system alliance 2.7 (UVitec, Cambridge, UK) and quantified using the software alliance V_1607. The primary antibodies used were α‐p53 (FL393‐G, Santa Cruz Biotechnology, Dallas, TX, USA) and α‐vinculin (V284, Millipore). The IgG–HRP–conjugated secondary antibodies used were α‐goat (50‐101P, Bethyl Laboratories, Montgomery, TX, USA) and α‐mouse (1706516, Bio‐Rad).

### 2.10 Cell cycle analysis

For cell cycle profiling, 5 × 10^5^ HCT116 cells were collected at 96 and 102 h after treatment. Cells were washed with PBS, fixed with 70% ethanol, treated with 2 mg·mL^−1^ RNaseA, stained with 0.1 mg·mL^−1^ propidium iodide/PBS, and analyzed using FACS (BD FACSCanto™ II, Franklin Lakes, NJ, USA). Cell cycle analysis was performed using flowjo software (Becton Dickinson, Franklin Lakes, NJ, USA).

### 2.11 Lentivirus production and transduction of target cells

Lentivirus was produced in HEK 293TX cells grown in DMEM high glucose supplemented with 10% FBS (Corning) and 1% penicillin–streptomycin (Sigma‐Aldrich). The lentiviral packaging vectors (pMDL‐GagPol, pRSV‐Rev, and pIVS‐VSV‐G) were cotransfected with the gene of interest (pcDNA‐LUC) using JetPrime reagent (Polyplus). At 48 and 72 h after transfection, the viral supernatant was collected and filtered using a 0.45‐μm cell strainer to remove cell debris. Virus particles were concentrated using a Lenti‐X Concentrator (Clontech, Takara) and resuspended in 1× DPBS. HCT116 cells were infected with a virus MOI 3 with 10 μg·μL^−1^ of polybrene (Sigma‐Aldrich) and selected using 1 μg·mL^−1^ puromycin (Sigma‐Aldrich).

### 2.12 Mice

Animal studies obtained ethical approval from the Ministry of Health (Protocol N° 79/2023‐PR) and were conducted conforming to the institutional guidelines in compliance with named Italian legislation (DL N116, GU, suppl. 40, 18‐2‐1992). Inbred BALB/c nude mice (CAnN.Cg‐Foxn1^nu^/J) obtained from the CNR‐IBBC‐EMMA‐Infrafrontier animal facility were housed in two per cage in the CNR‐IBBC‐EMMA‐Infrafrontier animal house. All animals were housed under controlled lighting conditions (daily light period, 07.00–19.00 h) at 20 ± 2° C, with *ad libitum* access to food (Emma 23, Mucedola, Settimo Milanese, Italy) and chlorinated filtered water. Five‐ to seven‐week‐old Foxn1^nu^/Foxn^nu^ male mice were injected subcutaneously in both flanks with luciferase‐expressing HCT116 (3 × 10^6^ cells per tumor) resuspended in 10 mg·mL^−1^ matrigel (dilution 1 : 1; BD Biosciences) to induce tumor formation. Two mice received Pep3 or Pep3S in paired xenografts in the same mouse; three received Pep3S or DMSO in paired xenografts, and two received Pep3 or DMSO in paired xenografts. After detecting a palpable tumor (tumor volume ≥ 70 mm^3^), mice were treated with either Pep3 or Pep3S and DMSO as control and injected intratumorally at 10 mg·kg^−1^. Tumor growth was followed and analyzed by luciferase activity over time. Luciferin was injected subcutaneously and after 15′ images were acquired. Bioluminescence acquisitions were performed with a Hybrid OI/CT system (MILabs, Houten, The Netherlands), and the images were analyzed using milabs‐oi‐pp software. For each tumor, the bioluminescence signal was normalized to the levels at the starting point.

### 2.13 ADMET prediction

The canonical SMILES of KVFIA (Pep3S) were generated using the PepSMI tool in NovoPro (https://www.novoprolabs.com). The SMILES string was used to predict the ADMET profile of Pep3S using SwissADME and pkCSM online tools [24, 25].

### 2.14 PLGA nanoparticle preparation and characterization

4.17 μL of a 10 mm stock solution of peptide in DMSO (Pep3S, Scramble3S, or FITC‐tagged Pep3S) or solvent DMSO was diluted with 1.67 mL of 0.31% wt (weight of solute/weight of solvent) of PLGA (Resomer® RG 505: poly(d, l‐lactide‐*co*‐glycolide) with molecular weight range 54–69 kDa) in the same solvent to obtain a 25 mm peptide concentration. 1.67 mL of this solution was mixed with 8.33 mL of a 0.015% wt Pluronic F127 (Sigma‐Aldrich) in deionized water under continuous vortexing and continuing vortexing for an additional 20″.

For *in vitro* and *in vivo* use, the resulting nanoparticle dispersion was dialyzed in a Spectra‐Por® Float‐A‐Lyzer® (MWCO 20 kDa) against 1 L of PBS 1× at room temperature for 3 h. Then, PLGA nanoparticles (NPs) were filtered through a 0.2 μm cellulose acetate (CA) syringe filter to sterilize them and eliminate potential aggregates.

For quantification and characterization, the nanoparticle dispersion was dialyzed against 1 L of Milli‐Q water for 3 h, then filtered with a 0.2 μm CA syringe filter, and checked by Dynamic Light Scattering.

### 2.15 Dynamic light scattering (DLS)

Hydrodynamic size and polydispersity (PDI) were measured at 25 °C using a Mobius instrument (Wyatt Technology, Dernbach, Germany) equipped with a 532 nm laser operating at a scattering angle 163.5°. Acquisition time was set at 5″ and 15″, respectively. Correlation functions were analyzed using the Dynals algorithm.

### 2.16 Fluorescamine assay

To quantify peptide loading in PLGA‐NPs, 1 mL of the nanoparticle's suspension (obtained as described above) was freeze‐dried. The pellet was dissolved in 300 μL of DMSO and used to quantify the peptide by fluorescamine assay. Briefly, 75 μL of the dissolved pellet in DMSO was transferred to a 96 black well plate and then mixed with 75 μL of a freshly made 1 mg·mL^−1^ fluorescamine in DMSO (maintained in a glass flask). The mixture was incubated for 5 min in the dark, and the fluorescence intensity (ex. 390 nm, em. 460 nm) was immediately measured using a BIOTek Synergy H1 multi‐mode microplate reader (BioTek Instruments, Inc., headquartered in Winooski, VT, USA). A mixture of PLGA and Pluronic equivalent to that present in the NP was used as a blank. The experimental loading was calculated by comparing the values obtained with the free peptide's calibration curve. The experiment was performed in triplicate.

### 2.17 Asymmetric flow field flow fractionation (AF4) and nanoparticle tracking analysis (NTA)

AF4 analyses were performed using an AF4 system AF2000 TM (Postnova Analytics) coupled online to a UV/VIS detector operating at 280 nm (Shimadzu SPD‐20A, Postnova Analytics, Landsberg am Lech, Germany), a fluorescence detector operating with an excitation wavelength at 490 nm and an emission one at 525 nm (Shimadzu RF‐20A), and finally a PN3609 multi‐angle light scattering (MALS) (Postnova Analytics) and PN3150 refractive index (Postnova Analytics) detectors in the given order. The AF4 frit‐inlet channel was equipped with a 350 μm spacer and a 10 kDa MWCO membrane of regenerated cellulose as an accumulation wall. Milli‐Q water supplemented with 0.02% (w/v) NaN_3_ was filtered through a 0.1 μm filter and used as an eluent. In a typical experiment, the detector flow rate was set at 0.5 mL·min^−1^, and 50 μL of sample was injected, setting 0.1 mL·min^−1^ as injection flow rate. For the elution step, the crossflow was maintained constant at 1 mL·min^−1^ for 2 min and then exponentially (exponent = 0.20) decreased to 0.1 mL·min^−1^ over 40 min. Subsequently, it was kept at 0.1 mL·min^−1^ for an additional 5 min. Last, a rinse step (i.e., cross flow at 0 mL·min^−1^ and purge valve on) was performed for 5 min. Each sample was analyzed 3 times. The data were analyzed using af2000 software (Postnova Analytics) and fitted with a sphere model assuming a dn/dc value of 0.165 mL·g^−1^, obtaining mass and radius of gyration distributions.

NTA analyses were performed by diluting 1 : 100 0.20 mg·mL^−1^ nanoparticle dispersions in Milli‐Q water using a NanoSight NS300 instrument (Malvern Panalytical, Malvern, UK) using a flow index of 50.

### 2.18 Statistical analysis

Statistical analysis was performed using the prism v8.0.2 (GraphPad, Boston, MA, USA) software. Statistically significant differences are indicated (*P ≤ 0.05, **P ≤ 0.01, ***P ≤ 0.001, and ****P ≤ 0.0001). Differences between the groups were considered significant at a *P* value ≤ 0.05. Unless indicated otherwise, all data are presented as mean ± SD or SEM.

## 3 Results

### 3.1 Pep3S reduces tumor cell growth *in vitro* and *in vivo*

**Fig. 1:**
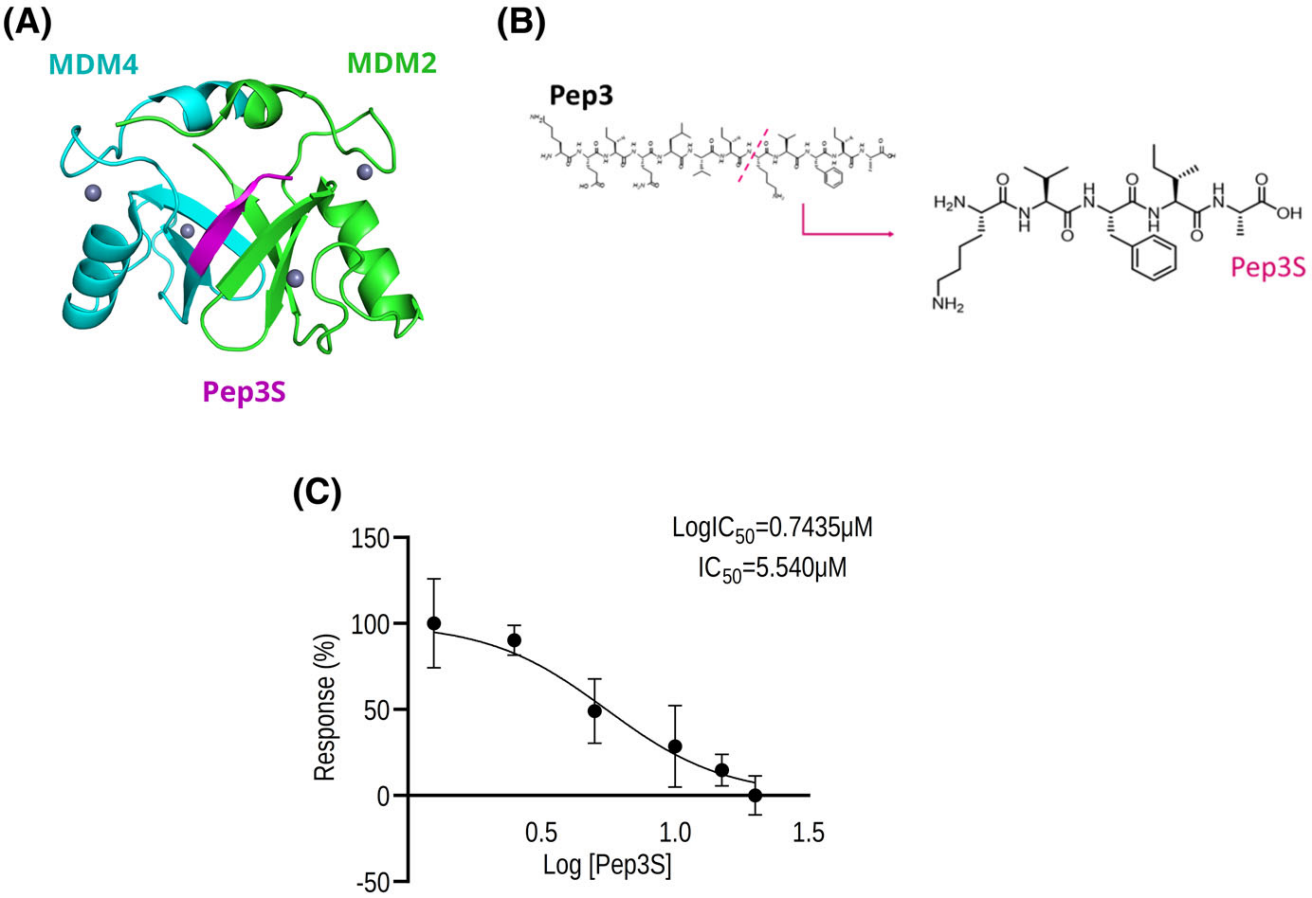
Features of the shortened peptide, Pep3S. (A) Putative binding model of Pep3S to the interaction interface of the MDM2/MDM4 C‐terminal RING (Really Interesting New Gene) domains (PDB ID: 2VJF). The green structure refers to MDM2, the light blue to MDM4, and the magenta to Pep3S. (B) The primary structure of Pep3 and derivative Pep3S, which encompasses the last five amino acids. (C) Dose–response curve of Pep3S in 2D HCT116 cells after 96 h. Data are from three independent experiments and is shown as mean ± SD.

The short peptide Pep3S, composed of the last five amino acids of the COOH‐terminus of MDM4 (Fig. 1A,B), possesses a higher *in vitro* affinity to MDM2 compared to the long peptide Pep3 (0.152 ± 0.007 vs. 0.245 ± 0.029 nm, respectively) and to a control peptide composed of the same amino acids but with a different sequence (Scramble3S, VAIKF) (0.447 nm ± 0.028).

Therefore, we evaluated whether this higher affinity is associated with increased biological activity.

**Fig. 2:**
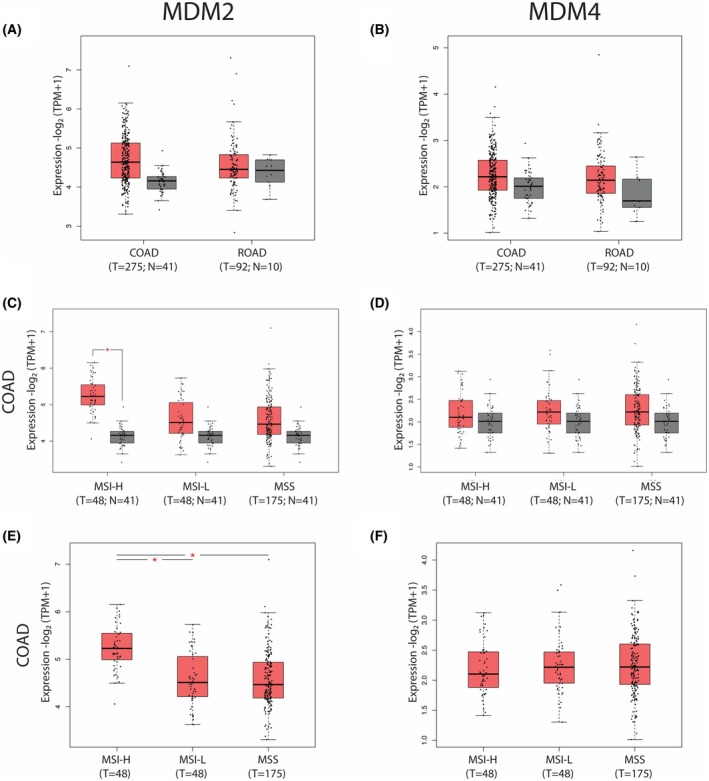
MDM2 and MDM4 transcript levels in tumors from Cancer Genome Atlas Program (TGCA) vs. TCGA normal tissue by Gene Expression Profiling Interactive Analysis (GEPIA2). (A, B) MDM2 and MDM4 transcript levels in colon adenocarcinoma (COAD) and rectum adenocarcinoma (READ) compared to paired normal samples, with each dot representing a distinct tumor or normal sample. The number of samples is reported for T (tumor sample) and N (normal sample). (C–F) MDM2 and MDM4 transcript levels in COAD subtypes: high microsatellite instability (MSI‐H), low microsatellite instability (MSI‐L), and microsatellite stability (MSS). Levels were compared to paired normal samples (C, D) or among the subtypes (E, F). Transcript levels are expressed as lg_2_(TPM + 1). The applied lg_2_ fold change was > 0.58. Data are represented by box plot showing the median and CI. *P* values were calculated by one‐way ANOVA, using disease state (Tumor or Normal) as variable for calculating differential expression. *P ≤ 0.05.

Analysis of IC50 showed a value of 5.54 μm for Pep3S (Fig. 1C) compared to 10 μm for Pep3 [20], supporting Pep3S higher efficiency. To test Pep3S's tumor‐suppressive properties, we focused on colorectal cancer (CRC) cell lines. The frequency of the wild‐type (wt) p53 gene in CRC is approximately 40%, which offers a potential window for the therapeutic application of p53‐reactivating molecules. In public datasets, MDM2 levels, whose overexpression is inversely correlated with p53 mutation in CRC [26], are increased in Colon adenocarcinoma (COAD) and not in Rectum adenocarcinoma (READ) compared to paired healthy tissue (Fig. 2A). The MDM4 levels showed a similar trend in COAD without reaching statistical significance (Fig. 2B). In the COAD group, MDM2, but not MDM4, levels were significantly upregulated in tumors with high microsatellite instability (MSI‐H), a histotype with low p53 mutation frequency [27] (Fig. 2C–F).

**Fig. 3:**
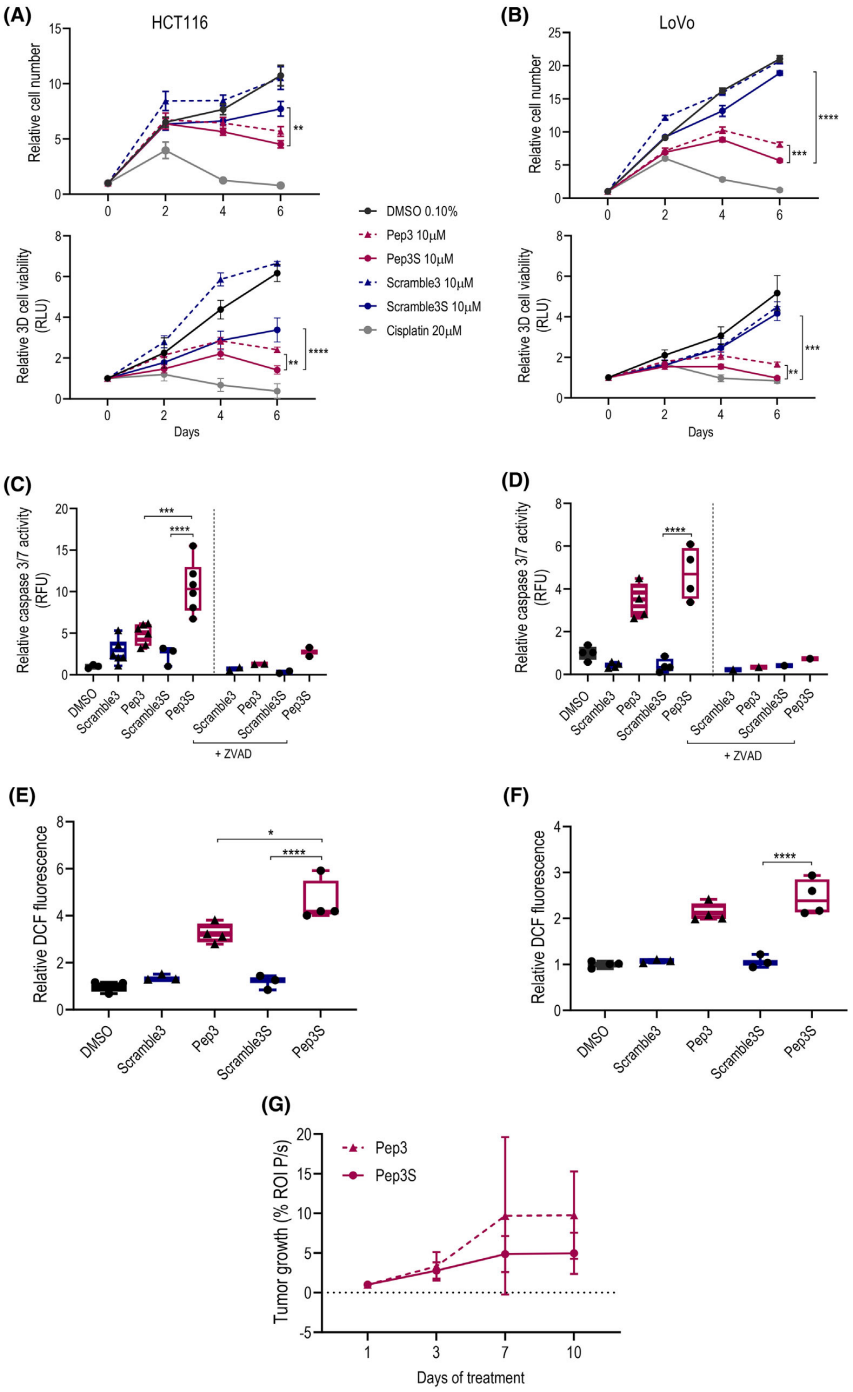
Pep3S reduces cell viability and increases apoptosis and oxidative stress in wild‐type (wt) p53 colorectal cancer (CRC) cell lines. (A, B), Viability assay in 2D (upper panel) and in 3D (lower panel) cultures of HCT116 (A) and LoVo (B) cells treated as indicated. HCT116 data are pooled from six (2D) and three (3D) independent experiments; LoVo data are pooled from three independent experiments. (*n* = 3/treatment/experiment) (C, D), Caspase 3/7 activity assay in 3D HCT116 (C) and LoVo (D), after 6 days of indicated treatments. The fluorescence signal was normalized to total protein, and data were normalized to DMSO levels set to 1. The caspase inhibitor ZVAD was used as a control. Symbols represent independent biological replicates (*n*_HCT116_ = 6/treatment; *n*_LoVo_ = 4/treatment; *n*_ZVAD_ = 2/treatment). (E, F), Analysis of reactive oxygen species accumulation in 2D HCT116 (E) and LoVo (F) after 48 h of indicated treatments. The dichlorofluorescein (DCF) fluorescence intensity was normalized to the number of cells/well. Data are normalized to DMSO levels set to 1. Symbols represent biological replicates (*n* = 4/treatment). (G) Quantification of bioluminescence signal (region of interest photon/s, ROI P/s) of HCT116‐luc from tumor xenograft 10 days after subcutaneous injection. Bioluminescence signal was detected at 1, 3, 7, and 10 days following 10 mg·kg^−1^ Pep3S or Pep3 intratumor treatment. Two mice received Pep3 or Pep3S in paired xenografts of the same mouse; three received Pep3S or DMSO in paired xenografts, and two received Pep3 or DMSO in paired xenografts (*n*_Pep3_ = 3; *n*_Pep3S_ = 5). All graphs show the mean ± SD. *P* values were calculated by two‐way ANOVA Tukey's multiple comparison test (A, B) and one‐way ANOVA Tukey's multiple comparison test (C, G). *P ≤ 0.05, **P ≤ 0.01, ***P ≤ 0.001, and ****P ≤ 0.0001.

Pep3S significantly reduced the growth of the MSI‐H HCT116 (Fig. 3A), and LoVo cells (Fig. 3B) in both 2D and 3D culture models compared to the respective negative control scrambled peptides (Scramble3S or Scramble3) and vehicle (DMSO). In most assays, it was more efficacious than Pep3 (Fig. 3A,B). Pep3S activity was also maintained in the long‐term cell growth assays (Fig. S1A,B). Pep3S showed p53 specificity, as evidenced by null effects in p53^−/−^HCT116 cells (Fig. S1C,D). Analysis of caspase‐3/7 activity in 3D models indicated significant pro‐apoptotic activity of Pep3S (Fig. 3C,D), confirmed by Propidium iodide (PI)‐positive cells in 3D spheroids (Fig. S2A,B), especially in the intermediate corona zone under the outermost proliferating layer (Fig. S2A,B). The pro‐apoptotic activity of Pep3 has previously been linked to the activation of an oxidative stress response [20]. Indeed, the short peptide Pep3S induced oxidative stress, which was higher than that resulting from the long peptide (Fig. 3E,F).

*In vivo*, Pep3S reduced the tumor growth of subcutaneous (sc) xenografts more efficiently than Pep3 (Fig. 3G).

Thus, Pep3S's high affinity for MDM2 improves the overall efficacy of MDM2/MDM4 heterodimer targeting.

### 3.2 Pep3S activates p53‐mediated transcriptional repression

**Fig. 4:**
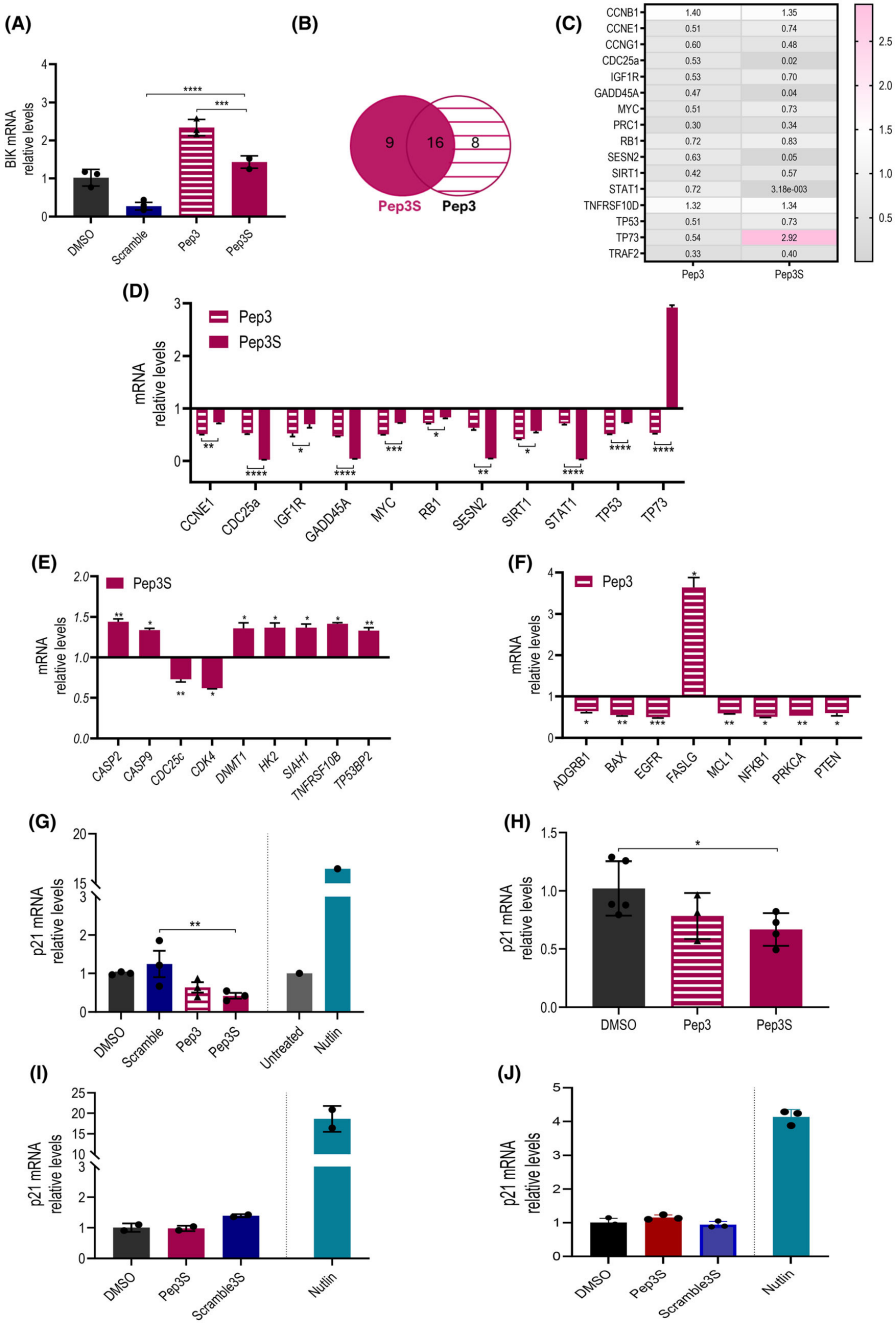
Pep3S activates p53‐mediated transcriptional repression. (A) BIK mRNA relative levels by quantitative reverse transcription polymerase chain reaction (RT‐qPCR) in HCT116 cells treated as indicated for 48 h (*n* = 3 for each treatment). (B) Venn diagram of the 33 differentially expressed genes (DEGs) significantly modulated by Pep3S (9/33) and Pep3 (8/33) or both (16/33) compared to a Scramble mix (Scramble3 plus Scramble3S). (C) Heat map of the 16/33 DEGs significantly modulated by both peptides compared to Scramble mix. (D) Group of the 11/16 DEGs (represented in the heat map) differently modulated by Pep3S and Pep3 compared to Scramble mix. (E, F) DEGs significantly modulated by only Pep3S (E) or Pep3 (F) compared to Scramble mix. (G) p21 mRNA relative levels by RT‐qPCR in HCT116 cells treated as indicated for 48 h. Nutlin‐3a (Nutlin) was added as control for p21 induction. Data are pooled from three independent experiments. (H) p21 mRNA relative levels by RT‐qPCR in xenograft tumors (*n*_DMSO_ = 5, *n*_Pep3_ = 3, *n*_Pep3S_ = 5). (I, J) p21 mRNA relative levels by RT‐qPCR in human myoblasts (I) or lymphoblastoid cells from healthy donors (J) treated as indicated for 48 h. Data are shown as mean ± SD (A–F; H) or ±SEM (G). *P* values were calculated by one‐way ANOVA Tukey's multiple comparison tests (A, E–G) and unpaired *t*‐test (H). *P ≤ 0.05, **P ≤ 0.01, ***P ≤ 0.001, and ****P ≤ 0.0001.

The pro‐apoptotic activity of Pep3 is mediated by the activation of the pro‐oxidant p53‐targets PIG3 and BIK [20]. BIK levels were increased by Pep3S. However, they were not higher than those in Pep3‐treated cells, even lower (Fig. 4A), suggesting that other p53 targets could mediate the increased efficacy of Pep3S. To understand the molecular pathways underlying Pep3S activity, we analyzed differentially expressed genes (DEGs) by Pep3S and Pep3 compared to a mix of Scramble3 and Scramble3S in HCT116 cells using a p53‐transcriptional array containing 80 validated targets of the human p53 pathway. Globally, 33 out of the 80 p53 targets were significantly altered by Pep3 and/or Pep3S compared to Scramble‐treated cells. Twenty‐five and 24 DEGs were modulated by Pep3S and Pep3, respectively, with 16 shared by both peptides (Fig. 4B,C). The expression levels of many of these common targets (11/16) varied significantly between Pep3 and Pep3S (Fig. 4D; asterisks under the bar). Conversely, 9/33 DEGs (27%) were unique to Pep3S (Fig. 4E and Fig. S3A) and 8/33 (24%) to Pep3 (Fig. 4F and Fig. S3B), supporting the hypothesis that there are differences in the activities of the two peptides. The most common genes showed the same trend except for TP73 (Fig. 4C,D). Unexpectedly, most of the shared DEGs (10/16) were downregulated by both peptides (Fig. 4C,D). Independent experiments confirmed the downregulation of some of these DEGs (Fig. S3C–E). Of particular interest is SESN2, which exerts cytoprotective effects against oxidative stress, especially in CRC [28], reinforcing the occurrence of a pro‐oxidant response elicited by Pep3 and, more potently, by Pep3S. In comparison, Nutlin‐3a showed opposite activity by upregulating the levels of some of these targets (Fig. S3C,D). In particular, the strong upregulation of SESN2 by Nutlin‐3a confirmed the induction of a different p53 signaling pathway by the two approaches (Fig. 4D and Fig. S3C).

Transcriptional repression is an uncommon feature of p53 oncosuppressive activity [29, 30]. Although some genes are directly repressed by p53 binding to their promoters (Myc, IGF1R), the repressive activity of p53 is mainly attributed to indirect mechanisms [30, 31]. One mechanism is the upregulation of the CDK inhibitor p21 and activation of the DREAM complex, which mediates the transcriptional repression of a subset of genes [32, 33] and is mainly associated with p53‐induced cell cycle arrest [33, 34]. In the p53 array used, the levels of p21 were not significantly altered. Independent experiments to detect all p21 mRNA isoforms showed significant downregulation of p21 by Pep3S compared to the vehicle and its scramble peptide in both HCT116 (Fig. 4G) and LoVo cells (Fig. S3F) and tumor xenografts (Fig. 4H). Although less severe, Pep3 caused a similar decrease (Fig. 4G,H and Fig. S3F). As a positive control, Nutlin‐3a strongly induced p21 expression (Fig. 4G and Fig. S3F). Consistent with these data, no substantial alterations in the cell cycle phases were detected, supporting the absence of cell growth arrest (Fig. S4). These data excluded the induction of DREAM‐mediated repression by Pep3S. The downregulation of RB1, a key component of the DREAM complex, further reinforces the exclusion of the DREAM pathway from the observed repression of DEGs (see Fig. 4C,D). The cell cycle arrest is a critical determinant of the toxicity of p53‐reactivating molecules due to the p53‐mediated depletion of nontumor cells [2]. We assessed the impact of Pep3S on healthy myoblasts, a tissue in which a faulty mechanism of skeletal muscle regeneration and repair contributes to cachexia, a debilitating condition affecting many advanced colorectal cancer patients [2, 35]. At odds with Nutlin‐3a, Pep3S did not affect p21 levels (Fig. 4I), although it was efficiently internalized like in HCT116 cells (Fig. S5A) and did not alter cell proliferation. Similarly, Pep3S was ineffective in reducing p21 levels and cell proliferation of immortalized lymphoblastoid cells (LCLs) from healthy donors [23], whereas Nutlin‐3a was highly potent (Fig. 4J and Fig. S5B). These data confirm the absence of a growth arrest response raised by Pep3S and support the hypothesis of reduced toxicity of this p53‐reactivating approach in healthy proliferating cells.

**Fig. 5:**
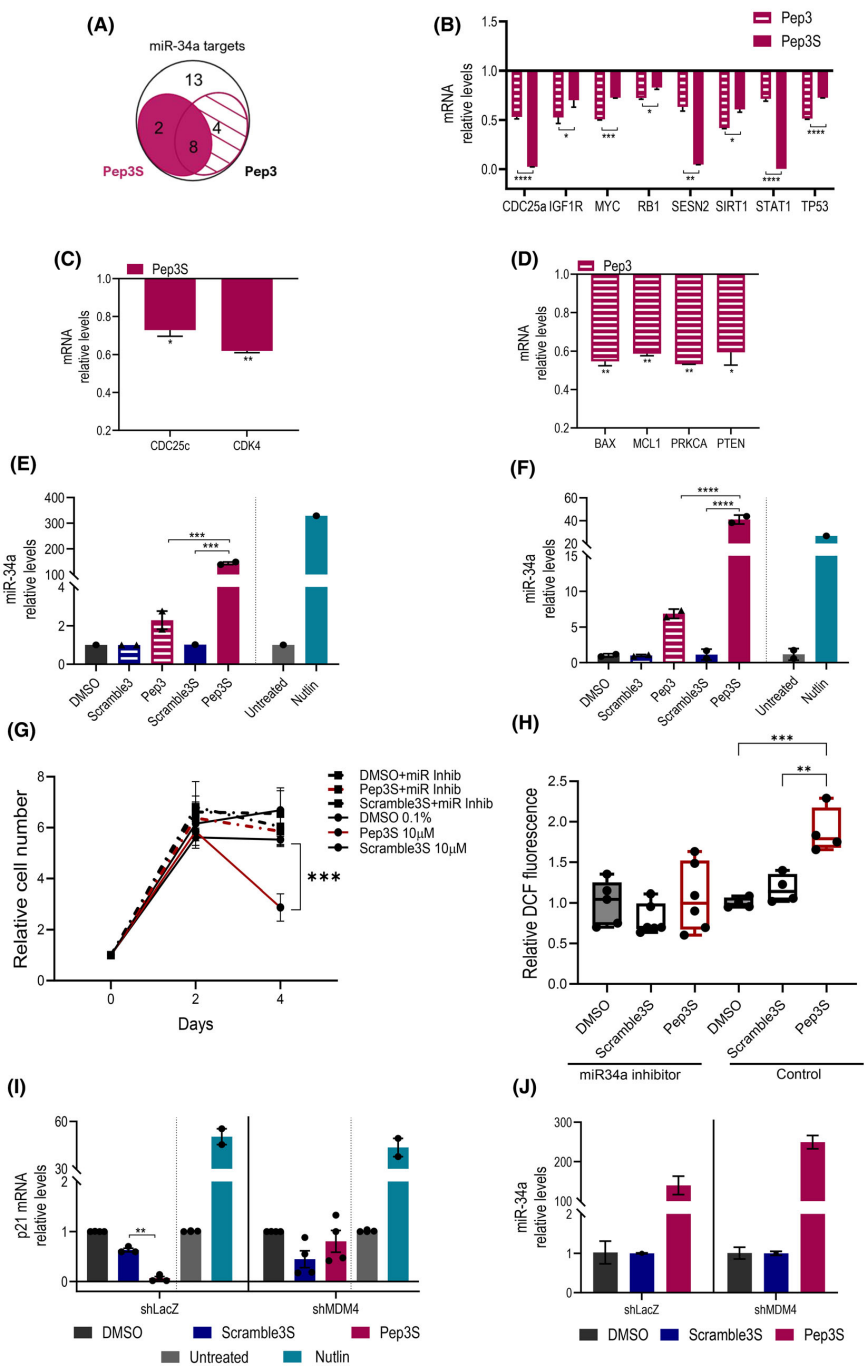
A subset of p53 differentially Expressed Genes (DEGs) are miR‐34a targets. (A) Venn diagram of the Pep3S‐ and Pep3‐downregulated DEGs and miR‐34a targets (identified by miRGator v3.0, miRTarBase, and TargetScanHuman). Eight out of 27 DEGs were downregulated by both, whereas 2/27 and 4/27 were downregulated by only Pep3S and Pep3, respectively. (B) Levels of miR‐34a targets modulated by both peptides (asterisks under the bar refer to the comparison between Pep3S and Pep3). (C, D) Levels of miR‐34a targets significantly modulated by Pep3S (C) or Pep3 (D) compared to Scramble. (E, F) MiR‐34a relative expression levels by RT‐qPCR in HCT116 (E) and LoVo (F) cells treated as indicated for 48 h. Data are a pool of two independent experiments performed with two biological replicates. (G, H) Viability assay (G) and reactive oxygen species accumulation (H) in 2D HCT116 cells transfected with miR‐34a inhibitor or control and treated as indicated. The dichlorofluorescein (DCF) fluorescence intensity was normalized to the number of cells/well. Data are from six independent biological replicates for miR‐34a inhibitor and four replicates for control. (I, J), Relative mRNA levels of p21 (I) and miR‐34a (J) by RT‐qPCR in HCT116 following silencing of MDM4 (shMDM4) or control (shLacZ) after 48 h of indicated treatments. Data are from two independent experiments with two biological replicates. Data are shown as mean ± SD (B–F, G, H) or ± SEM (I). *P* values were calculated using one‐way ANOVA Tukey's multiple comparison test (C–J). *P ≤ 0.05, **P ≤ 0.01, ***P ≤ 0.001, and ****P ≤ 0.0001.

Another indirect mechanism of p53‐mediated repression involves the induction of miR‐34a. This miRNA is an important oncosuppressor that mediates the transcriptional repression of various targets and is induced by p53, triggering a pro‐apoptotic response [36, 37, 38]. Comparative analysis revealed that 8 of the 10 genes commonly downregulated by Pep3S and Pep3 (Fig. 4D) were also targets of miR‐34a (Fig. 5A,B). In addition, 2/2 and 4/7 of the p53 targets downregulated by Pep3S and Pep3 (Fig. 4E,F), respectively, were targets of miR‐34a (Fig. 5A,C,D). Analysis of miR‐34a revealed a significant induction of its levels by Pep3S and, to a lesser extent, Pep3 in both HCT116 and LoVo cells (Fig. 5E,F). Nutlin‐3a induced miR‐34a expression, as well as previously described [39]. The lack of miR‐34a activation in p53^−/−^ HCT116 cells (Fig. S6A) proved the p53‐dependency in the activation of this miRNA.

To understand the relevance of miR‐34a in Pep3S efficacy, we inhibited miR‐34a activity with a specific mRNA inhibitor (Fig. S6B). The inhibitor impaired both cell viability and oxidative stress response (Fig. 5G,H), indicating that the miR‐34a is relevant for the oxidative stress response and the loss of cell viability raised by Pep3S.

Therefore, targeting the MDM2/MDM4 heterodimer activates a specific p53 signature characterized by repression of many genes. Pep3S appeared to potentiate and simultaneously distinguish the signature underlying its efficacy compared to the long peptide.

### 3.3 Activation of p53/p21 pathway is mediated by MDM4

Pep3S activity appears to be associated with two distinct molecular features: the downregulation of p21 and the upregulation of miR‐34a. While the upregulation of miR‐34a is common to other p53 activating molecule, the downregulation of p21 mRNA appears to be a unique feature of MDM2/MDM4 heterodimer targeting, particularly by Pep3S. Therefore, we investigated how the heterodimer target is responsible for this result. Previous studies have shown that MDM4 inhibits p53‐mediated activation of p21 independently of MDM2 [15, 16, 40, 41, 42]. Because heterodimer dissociation leaves the single MDM4 protein free to interact with p53, we tested the effect of Pep3S‐dissociated MDM4 on p21 repression. Doxycycline‐inducible MDM4 silencing (Fig. S6C) prevented the downregulation of p21 compared to that observed in shLacZ‐interfered cells (Fig. 5I). In contrast, it was ineffective in the upregulation mediated by Nutlin‐3a (Fig. 5I). In the absence of peptide treatment, shMDM4 did not substantially alter p21 expression (Fig. S6D), indicating that it is the dissociated protein to repress p53 activity. In p53^−/−^HCT116 cells, Pep3S treatment did not alter p21 levels independently of the presence of MDM4 (Fig. S6E), proving that p21 levels are decreased by Pep3S in an MDM4/p53 crosstalk‐dependent manner. Conversely, the upregulation of miR‐34a levels was not altered by shMDM4 and was even reinforced (Fig. 5J).

These data demonstrate that Pep3S‐mediated release of MDM4 induces a crosstalk between p53 and MDM4, which downregulates p21.

### 3.4 PLGA‐Pep3S formulation improves Pep3S efficacy

Previous data suggest the potential therapeutic efficacy of Pep3S in CRC. Administration and stability are crucial factors for the therapeutic application of peptides, including Pep3S. *In silico* prediction of the absorption, distribution, metabolism, excretion, and toxicity (ADMET) properties of Pep3S indicated that this peptide has low oral bioavailability (resulting from the violation of the 3/5 Lipinski rules) and poor intestinal absorption (Table 1).

**Table 1:** *In silico* ADMET prediction of Pep3S.

Molecular descriptors		Absorption		Distribution		Metabolism		Toxicity	
Property	Predicted value	Property	Predicted value	Property	Predicted value	Property	Predicted value	Property	Predicted value
Molecular weight	576.73	Intestinal absorption	Low (SwissADME) 8.767 (pkCSM)	BBB permeability	No (SwissADME) −0.781 (pkCSM)	CYP1A2 inhibitor	No (SwissADME and pkCSM)	Ames toxicity	No (pkCSM)
Log *P*	0.77 (SwissADME) 0.4312 (pkCSM)	Caco‐2 permeability	0.059 (pkCSM)			CYP2C19 inhibitor	No (SwissADME and pkCSM)		
#Rotatable bonds	22 (SwissADME) 18 (pkCSM)	P‐glycoprotein substrate	Yes (Swiss ADME) Yes (pKCSM)			CYP2C9 inhibitor	No (SwissADME and pkCSM)		
#H‐bond acceptors	8 (SwissADME) 7 (pkCSM)					CYP2D6 inhibitor	No (SwissADME and pkCSM)		
#H‐bond donors	7 (SwissADME) 7 (pkCSM)					CYP3A4 inhibitor	No (SwissADME and pkCSM)		
Polar surface area	205.74 (SwissADME) 242.306 (pkCSM)								
Lipinski #violations	3								

Therefore, we considered the potential benefits of Pep3S encapsulation in nanoparticles. Poly (lactic‐co‐glycolic acid) (PLGA) nanoparticles are FDA‐ and EMA‐approved tools already used to deliver peptide entities, among others [43]. PLGA was loaded with 1% w/w Pep3S, FITC‐Pep3S, Scramble3S, or DMSO solvent as control, and their properties were analyzed. Dynamic light scattering (DLS) showed that the diameter of PLGA‐Pep3S nanoparticles was within a 40–60 nm range with a low polydispersity (PDI) mean value, excluding the potential undesired clearance process (Fig. S7 and Table S2). Asymmetric field flow fractionation used to estimate the weight mass of these nanoparticles indicated they were within the therapeutic range (Table S3). Particle tracking analysis by nanoparticle tracking analysis (NTA) estimated that the PLGA‐Pep3S formulation contained 2.12e + 10 ± 6.50e + 08 particles·mL^−1^, with Pep3S loading about 2.6 μm (Table S2).

**Fig. 6:**
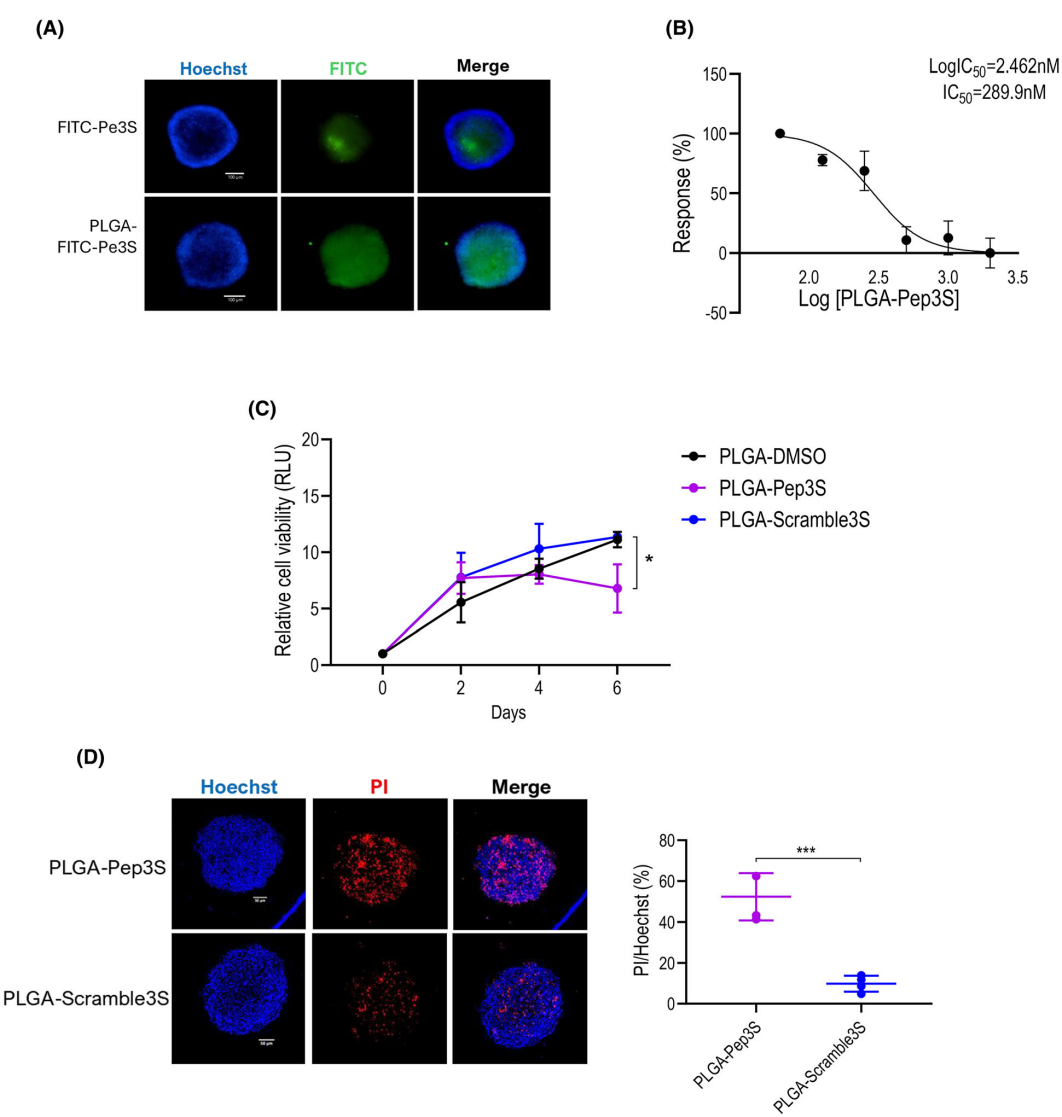
Poly (lactic‐co‐glycolic acid) (PLGA) nanoparticles increased Pep3S efficacy. (A) Representative fluorescence images of HCT116 spheroids 48 h following a single treatment with PLGA‐FITC‐Pep3S or naked FITC‐Pep3S. Scale bars, 100 μm. (B) PLGA‐Pep3S dose–response curve. Data are from three biological replicates. (C) Viability assay of HCT116 spheroids treated as indicated. Data are from four independent experiments with three spheroids for treatment. *P* value was calculated by unpaired *t*‐test. (D) Representative confocal images after 6 days of indicated treatment. Scale bar, 50 μm. The graph on the right is representative of two independent experiments with three replicates (*n* = 3/experiment). The percentage of the PI signal was measured through imagej and normalized to the Hoechst signal. Data are shown as mean ± SD. *P* value was calculated by unpaired *t*‐test; *P ≤ 0.05, ***P ≤ 0.001.

Compared to naked FITC‐tagged Pep3S, PLGA‐FITC‐Pep3S showed increased and more diffused fluorescence in 3D tumor spheroids (Fig. 6A), proving that PLGA encapsulation increases cell penetrance and peptide stability. Biological testing of PLGA‐Pep3S on 3D spheroids indicated an IC_50_ of 0.289 μm (Fig. 6B), a 20‐fold increase over the nude peptide (see Fig. 1C). PLGA‐Pep3S reduced cell growth (Fig. 6C) and caused marked apoptosis (Fig. 6D), indicating that the PLGAs maintained and reinforced the biological activity of the nude peptide. Thus, Pep3S formulation in PLGA envisages a potentially helpful therapeutic application.

**Fig. 7:**
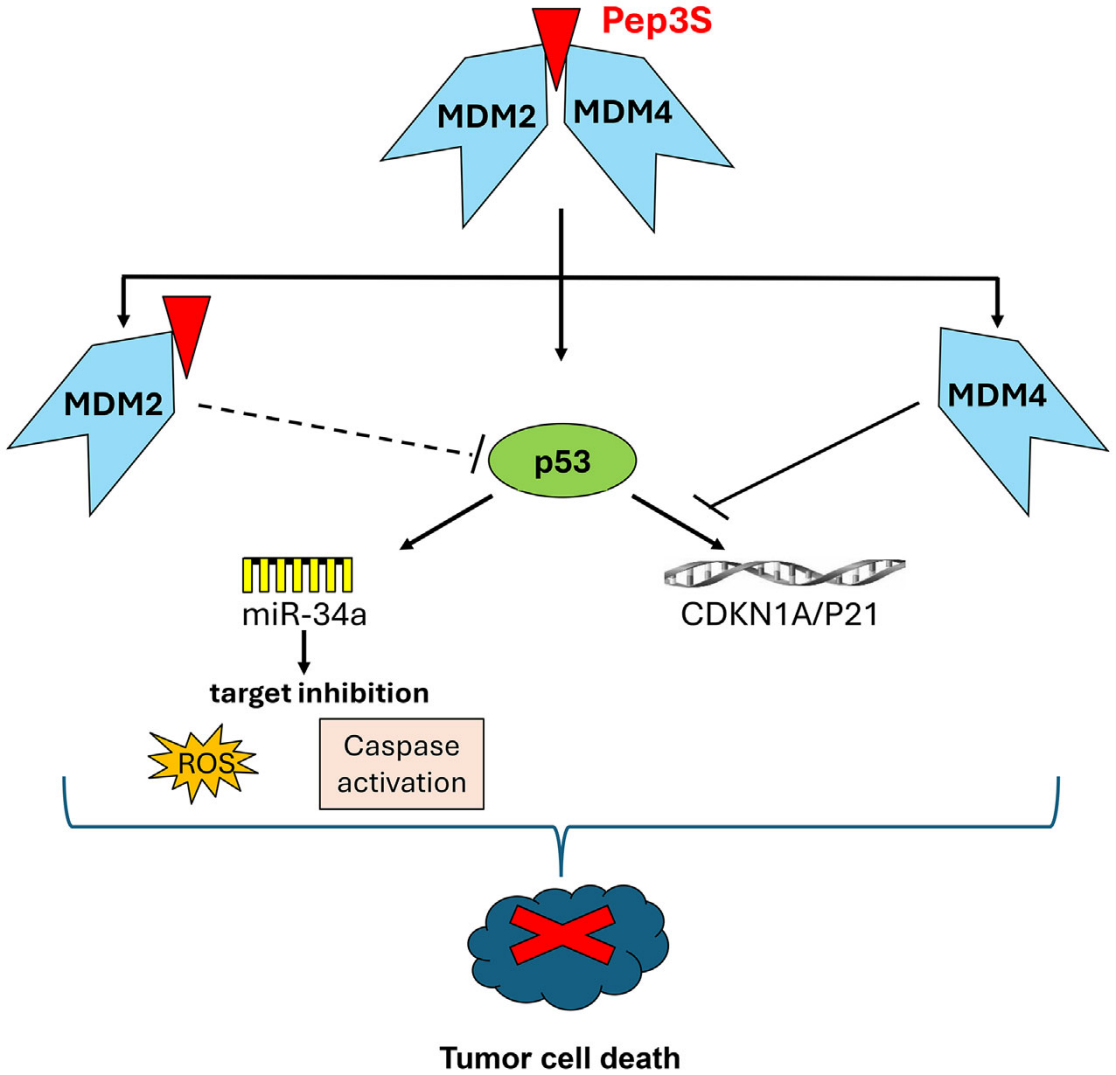
Scheme of Pep3S activity. Pep3S (the red triangle) binds MDM2 leading to heterodimer dissociation. This reduces p53 inhibition by MDM2. Activated p53 induces miR‐34a expression which, by inhibiting its targets, causes oxidative stress and caspase activation. Dissociated MDM4 inhibits p53‐mediated activation of CDKN1A/P21.

Overall, these data prove the efficacy of targeting the heterodimer MDM2/MDM4 by a short peptide, which mediates an otherwise p53 activation with potentially reduced toxicity in nontumor tissues (Fig. 7).

## 4 Discussion

The short peptide described in this work efficiently induced p53 activity *in vitro* and *in vivo* and proves the therapeutic usefulness of targeting the MDM2/MDM4 heterodimer binding region.

Activating cell death response is the gold standard for any therapeutic approach. Many of the molecules developed to reactivate p53 oncosuppressive function activate cell death and cell cycle arrest in tumor cells. Nonetheless, in clinical trials, several of these molecules have shown toxicities that are often related to hypoplasia of proliferating tissues. A critical target of p53 activity is p21, which acts as a relevant proliferation brake. Although p21 levels are not a determinant of the p53 response to Nutlin in tumor cells [44], they may represent a crucial factor in the depletion of nontransformed cells [9].

Based on a database that integrates datasets of regulatory mechanisms and networks of p53 and p53‐dependent gene regulation [45], all molecules involved in p53 reactivation imply p21 mRNA upregulation [46]. To our knowledge, Pep3S is the first molecule to deviate from this trend, significantly downregulating p21 mRNA levels more efficiently than Pep3. Since Pep3S did not affect the proliferation status of nontransformed muscle and lymphoblastoid cells, these data support and reinforce the potentially reducing toxicity of this approach.

Among other p53‐activating molecules, MMRi64, a molecule targeting the MDM2/MDM4 heterodimer, showed minimal induction of p21 followed by its strong downregulation [21], supporting the association of this unique feature with heterodimer impairment. P21 has also shown anti‐apoptotic activity [47], which could contribute to antagonizing the p53‐mediated apoptotic response. Therefore, the downregulation of p21 might also be beneficial for tumor suppression.

A previous molecule, RITA, also caused the downregulation of p21 protein levels (43), which was associated with the activation of the apoptotic response, reinforcing the previous hypothesis.

The downregulation of p21 appears to be integrated with a predisposing pro‐apoptotic signature through the induction of pro‐oxidant genes such as BIK and an increase in miR‐34a. P53 induces different miRNAs [48]. Compared to other miRNAs, the induction of miR‐34 has been indicated as a switch towards cell death, given the pro‐apoptotic activity of this miRNA [37, 38]. Although the mechanism underlying this switch is unclear, strong activation of miR‐34a by Pep3S represents a favorable response for tumor suppression. Accordingly, we proved that the downregulation of this miRNA reduces oxidative stress levels and impairs cell viability loss. In contrast to p21, this miRNA was still induced following MDM4 silencing, indicating that MDM4 is irrelevant for its p53‐mediated induction. Accordingly, miR‐34a is also induced in Nutlin‐treated cells, in which the binding between MDM2 and MDM4 is not impaired, whereas their binding to p53 is inhibited, although with different efficiencies [49].

We recently demonstrated that targeting the MDM2/MDM4 heterodimer also increases the antitumor immune response [50]. Interestingly, miR‐34a downregulates PDL1 [51], thus providing a molecular basis for the additional tumor‐suppressive activity of Pep3S.

In addition to potentiating the regulation of p53 targets compared to the long peptide, Pep3S raises a unique p53 signature. An interesting feature of Pep3S activity is the unique upregulation of caspase 2 and 9 genes, which appears to reinforce the cell death response in agreement with increased efficacy.

The extent to which these additional events could be attributed to the dissociated proteins MDM4 or MDM2 remains to be ascertained. Indeed, the potential persistence of MDM2 and MDM4 binding to p53 represents a unique feature of p53 reactivating molecules. The impairment of the heterodimer still allows mild control of p53 levels, as indicated by the lack of an increase in p53 levels [20], which occurs after Nutlin‐3a or other p53‐activating molecules [4]. This is probably mediated by the binding of MDM2 to p53, which, although with reduced efficiency, controls the p53 protein levels. In addition, the additional activity of MDM4 towards p53 under these circumstances cannot be excluded. Moreover, the altered activity of MDM2 towards other factors and its missed increased levels, as occurs with other p53 activating molecules, may contribute to the apoptotic response. Indeed, MDM2 has shown anti‐apoptotic functions by downregulating some p53 pro‐apoptotic activators [41, 52].

A critical advantage of therapeutic peptides is their reduced risk of resistance mechanisms and increased specificity [53]. Indeed, the levels of mutations induced in the target molecule were lower than those raised by small molecules. Unfortunately, the acquisition of p53 mutations remains an adverse effect of this oncosuppressor activation therapy [54]. In this regard, a rapid and potent cell death response may reduce the p53 mutation burden.

Finally, efficient conjugation of Pep3S with PLGA provides a potential pharmaceutical formulation for this peptide. Peptide sensitivity to bloodstream proteases precludes the direct administration of this type of therapeutic molecule. The use of non‐natural amino acids can overcome this problem. Alternatively, nanocarriers have provided an efficient tool for protecting therapeutic peptides [43]. In addition, the possibility of decorating nanoparticles with tumor‐specific markers permits the preferential delivery of these therapeutic tools to the tumor site. A recent work on the same pentapeptide reported the relevance of N‐ and C‐terminal peptide modifications, adding another factor to increase peptide efficiency [55].

Overall, this work demonstrates the usefulness of targeting the association region of MDM2 and MDM4 with a short peptide, which provides an alternative route for reactivating p53 oncosuppressive activity in colorectal cancer.

## 5 Conclusions

This work demonstrated that targeting the association region of MDM2 and MDM4 with a short peptide represents an alternative and favorable approach compared to other p53‐activating molecules based on the dissociation of p53 from its inhibitors MDM2 and MDM4. Our data highlight a transcriptional signature partly overlapping with previous molecules with unique features that impact the apoptotic response and potentially reduce toxicity towards proliferating nontumor tissues. Finally, the Pep3S conjugation which PLGA provides an immediately valuable tool for overcoming problems related to peptide‐based therapy.

## Conflict of interest

The authors declare no conflict of interest. Pep3S and its PLGA formulation are under PCT patenting (Application Number: PCT/IB2024/057215).

## Author contributions

SV contributed to the conception and design of the study, performed research, data analysis and interpretation, writing – original draft, and writing–review. GM performed research, data analysis and writing–review. MA, FSac, and FSar. RM performed research and data analysis. MRA, FSac, CR, NT, and AP contributed to the development of methodology and data analysis, provided critical feedback. FM: contributed to the conception and design of the study, performed data analysis and interpretation, supervised the study, performed writing – original draft, writing–review. All authors read and approved the final manuscript.

## Peer review

The peer review history for this article is available at https://www.webofscience.com/api/gateway/wos/peer‐review/10.1002/1878‐0261.70006.

## Supporting information


**Fig. S1.** Pep3S reduces clonogenic cell ability.
**Fig. S2.** Pep3S increases cell death of CRC spheroids.
**Fig. S3.** Molecular activities of Pep3S.
**Fig. S4.** Cell cycle analysis.
**Fig. S5.** Pep3S activity in non‐tumor cells.
**Fig. S6.** Effects of p53 and MDM4 on Pep3S activity.
**Fig. S7.** Analysis of PLGA nanoparticles.
**Table S1.** Primers used for qRT‐PCR primers.
**Table S2.** Features of PLGA nanoparticles loaded with Pep3S or Scramble3S.
**Table S3.** Properties of PLGA nanoparticles loaded with vehicle (DMSO), Pep3S, Scramble3S, or FITC‐Pep3S.

## Data Availability

The data that support the findings of this study are available in the paper and Supporting Information. Additional data are available upon request.

## References

[1] Hassin O , Oren M . Drugging p53 in cancer: one protein, many targets. Nat Rev Drug Discov. 2023;22(2):127–144. 36216888 10.1038/s41573-022-00571-8 PMC9549847

[2] Tuval A , Strandgren C , Heldin A , Palomar‐Siles M , Wiman KG . Pharmacological reactivation of p53 in the era of precision anticancer medicine. Nat Rev Clin Oncol. 2024;21(2):106–120. 38102383 10.1038/s41571-023-00842-2

[3] Zhu H , Gao H , Ji Y , Zhou Q , Du Z , Tian L , et al. Targeting p53‐MDM2 interaction by small‐molecule inhibitors: learning from MDM2 inhibitors in clinical trials. J Hematol Oncol. 2022;15(1):91. 35831864 10.1186/s13045-022-01314-3 PMC9277894

[4] Vassilev LT , Vu BT , Graves B , Carvajal D , Podlaski F , Filipovic Z , et al. In vivo activation of the p53 pathway by small‐molecule antagonists of MDM2. Science. 2004;303(5659):844–848. 14704432 10.1126/science.1092472

[5] Haronikova L , Bonczek O , Zatloukalova P , Kokas‐Zavadil F , Kucerikova M , Coates PJ , et al. Resistance mechanisms to inhibitors of p53‐MDM2 interactions in cancer therapy: can we overcome them? Cell Mol Biol Lett. 2021;26(1):53. 34911439 10.1186/s11658-021-00293-6 PMC8903693

[6] Patton JT , Mayo LD , Singhi AD , Gudkov AV , Stark GR , Jackson MW . Levels of HdmX expression dictate the sensitivity of normal and transformed cells to Nutlin‐3. Cancer Res. 2006;66(6):3169–3176. 16540668 10.1158/0008-5472.CAN-05-3832

[7] Hu B , Gilkes DM , Farooqi B , Sebti SM , Chen J . MDMX overexpression prevents p53 activation by the MDM2 inhibitor Nutlin. J Biol Chem. 2006;281(44):33030–33035. 16905541 10.1074/jbc.C600147200

[8] Skalniak L , Surmiak E , Holak TA . A therapeutic patent overview of MDM2/X‐targeted therapies (2014–2018). Expert Opin Ther Pat. 2019;29(3):151–170. 30822185 10.1080/13543776.2019.1582645

[9] Lu M , Wang X , Li Y , Tripodi J , Mosoyan G , Mascarenhas J , et al. Combination treatment in vitro with Nutlin, a small‐molecule antagonist of MDM2, and pegylated interferon‐α 2a specifically targets JAK2V617F‐positive polycythemia vera cells. Blood. 2012;120(15):3098–3105. 22872685 10.1182/blood-2012-02-410712 PMC3471518

[10] Mascarenhas J , Lu M , Kosiorek H , Virtgaym E , Xia L , Sandy L , et al. Oral idasanutlin in patients with polycythemia vera. Blood. 2019;134(6):525–533. 31167802 10.1182/blood.2018893545 PMC6688433

[11] Tanimura S , Ohtsuka S , Mitsui K , Shirouzu K , Yoshimura A , Ohtsubo M . MDM2 interacts with MDMX through their RING finger domains. FEBS Lett. 1999;447(1):5–9. 10218570 10.1016/s0014-5793(99)00254-9

[12] Gu J , Kawai H , Nie L , Kitao H , Wiederschain D , Jochemsen AG , et al. Mutual dependence of MDM2 and MDMX in their functional inactivation of p53. J Biol Chem. 2002;277(22):19251–19254. 11953423 10.1074/jbc.C200150200

[13] Migliorini D , Danovi D , Colombo E , Carbone R , Pelicci PG , Marine JC . Hdmx recruitment into the nucleus by Hdm2 is essential for its ability to regulate p53 stability and transactivation. J Biol Chem. 2002;277(9):7318–7323. 11744695 10.1074/jbc.M108795200

[14] Chen L , Borcherds W , Wu S , Becker A , Schonbrunn E , Daughdrill GW , et al. Autoinhibition of MDMX by intramolecular p53 mimicry. Proc Natl Acad Sci U S A. 2015;112(15):4624–4629. 25825738 10.1073/pnas.1420833112 PMC4403185

[15] Parant J , Chavez‐Reyes A , Little NA , Yan W , Reinke V , Jochemsen AG , et al. Rescue of embryonic lethality in Mdm4‐null mice by loss of Trp53 suggests a nonoverlapping pathway with MDM2 to regulate p53. Nat Genet. 2001;29(1):92–95. 11528400 10.1038/ng714

[16] Steinman HA , Sluss HK , Sands AT , Pihan G , Jones SN . Absence of p21 partially rescues Mdm4 loss and uncovers an antiproliferative effect of Mdm4 on cell growth. Oncogene. 2004;23(1):303–306. 14712235 10.1038/sj.onc.1206925

[17] Di Conza G , Mancini F , Buttarelli M , Pontecorvi A , Trimarchi F , Moretti F . MDM4 enhances p53 stability by promoting an active conformation of the protein upon DNA damage. Cell Cycle. 2012;11(4):749–760. 22374672 10.4161/cc.11.4.19208

[18] Mancini F , Di Conza G , Pellegrino M , Rinaldo C , Prodosmo A , Giglio S , et al. MDM4 (MDMX) localizes at the mitochondria and facilitates the p53‐mediated intrinsic‐apoptotic pathway. EMBO J. 2009;28(13):1926–1939. 19521340 10.1038/emboj.2009.154 PMC2711189

[19] Zhu Y , Regunath K , Jacq X , Prives C . Cisplatin causes cell death via TAB1 regulation of p53/MDM2/MDMX circuitry. Genes Dev. 2013;27(16):1739–1751. 23934659 10.1101/gad.212258.112 PMC3759692

[20] Pellegrino M , Mancini F , Luca R , Coletti A , Giacche N , Manni I , et al. Targeting the MDM2/MDM4 interaction interface as a promising approach for p53 reactivation therapy. Cancer Res. 2015;75(21):4560–4572. 26359458 10.1158/0008-5472.CAN-15-0439

[21] Wu W , Xu C , Ling X , Fan C , Buckley BP , Chernov MV , et al. Targeting RING domains of Mdm2‐MdmX E3 complex activates apoptotic arm of the p53 pathway in leukemia/lymphoma cells. Cell Death Dis. 2015;6:e2035. 26720344 10.1038/cddis.2015.358 PMC4720891

[22] Tang Z , Kang B , Li C , Chen T , Zhang Z . GEPIA2: an enhanced web server for large‐scale expression profiling and interactive analysis. Nucleic Acids Res. 2019;47(W1):W556–W560. 31114875 10.1093/nar/gkz430 PMC6602440

[23] Sardina F , Valente D , Fattorini G , Cioffi E , Zanna GD , Tessa A , et al. New cellular imaging‐based method to distinguish the SPG4 subtype of hereditary spastic paraplegia. Eur J Neurol. 2023;30(6):1734–1744. 36815539 10.1111/ene.15756

[24] Pires DE , Blundell TL , Ascher DB . pkCSM: predicting small‐molecule pharmacokinetic and toxicity properties using graph‐based signatures. J Med Chem. 2015;58(9):4066–4072. 25860834 10.1021/acs.jmedchem.5b00104 PMC4434528

[25] Daina A , Michielin O , Zoete V . SwissADME: a free web tool to evaluate pharmacokinetics, drug‐likeness and medicinal chemistry friendliness of small molecules. Sci Rep. 2017;3(7):42717. 10.1038/srep42717 PMC5335600 28256516

[26] Nyiraneza C , Jouret‐Mourin A , Kartheuser A , Camby P , Plomteux O , Detry R , et al. Distinctive patterns of p53 protein expression and microsatellite instability in human colorectal cancer. Hum Pathol. 2011;42(12):1897–1910. 21665242 10.1016/j.humpath.2010.06.021

[27] Mei WJ , Mi M , Qian J , Xiao N , Yuan Y , Ding PR . Clinicopathological characteristics of high microsatellite instability/mismatch repair‐deficient colorectal cancer: a narrative review. Front Immunol. 2022;23(13):1019582. 10.3389/fimmu.2022.1019582 PMC9822542 36618386

[28] Ala M . Sestrin2 in cancer: a foe or a friend? Biomark Res. 2022;10(1):29. 35527284 10.1186/s40364-022-00380-6 PMC9080202

[29] Peuget S , Selivanova G . p53‐dependent repression: DREAM or reality? Cancer. 2021;13(19):4850. 10.3390/cancers13194850 PMC8508069 34638334

[30] Rinn JL , Huarte M . To repress or not to repress: this is the guardian's question. Trends Cell Biol. 2011;21(6):344–353. 21601459 10.1016/j.tcb.2011.04.002

[31] Fischer M . Census and evaluation of p53 target genes. Oncogene. 2017;36(28):3943–3956. 28288132 10.1038/onc.2016.502 PMC5511239

[32] Engeland K . Cell cycle arrest through indirect transcriptional repression by p53: I have a DREAM. Cell Death Differ. 2018;25(1):114–132. 29125603 10.1038/cdd.2017.172 PMC5729532

[33] Uxa S , Bernhart SH , Mages CFS , Fischer M , Kohler R , Hoffmann S , et al. DREAM and RB cooperate to induce gene repression and cell‐cycle arrest in response to p53 activation. Nucleic Acids Res. 2019;47(17):9087–9103. 31400114 10.1093/nar/gkz635 PMC6753476

[34] Sadasivam S , DeCaprio JA . The DREAM complex: master coordinator of cell cycle‐dependent gene expression. Nat Rev Cancer. 2013;13(8):585–595. 23842645 10.1038/nrc3556 PMC3986830

[35] Fearon K , Arends J , Baracos V . Understanding the mechanisms and treatment options in cancer cachexia. Nat Rev Clin Oncol. 2013;10(2):90–99. 23207794 10.1038/nrclinonc.2012.209

[36] He L , He X , Lim LP , De SE , Xuan Z , Liang Y , et al. A microRNA component of the p53 tumour suppressor network. Nature. 2007;447(7148):1130–1134. 17554337 10.1038/nature05939 PMC4590999

[37] Chang TC , Wentzel EA , Kent OA , Ramachandran K , Mullendore M , Lee KH , et al. Transactivation of miR‐34a by p53 broadly influences gene expression and promotes apoptosis. Mol Cell. 2007;26(5):745–752. 17540599 10.1016/j.molcel.2007.05.010 PMC1939978

[38] Georges SA , Chau BN , Braun CJ , Zhang X , Dobbelstein M . Cell cycle arrest or apoptosis by p53: are microRNAs‐192/215 and ‐34 making the decision? Cell Cycle. 2009;8(5):2–682. 19223766

[39] Braun CJ , Zhang X , Savelyeva I , Wolff S , Moll UM , Schepeler T , et al. p53‐responsive micrornas 192 and 215 are capable of inducing cell cycle arrest. Cancer Res. 2008;68(24):10094–10104. 19074875 10.1158/0008-5472.CAN-08-1569 PMC2836584

[40] Barboza JA , Iwakuma T , Terzian T , El‐Naggar AK , Lozano G . Mdm2 and Mdm4 loss regulates distinct p53 activities. Mol Cancer Res. 2008;6(6):947–954. 18567799 10.1158/1541-7786.MCR-07-2079 PMC2699947

[41] Mancini F , Pieroni L , Monteleone V , Luca R , Fici L , Luca E , et al. MDM4/HIPK2/p53 cytoplasmic assembly uncovers coordinated repression of molecules with anti‐apoptotic activity during early DNA damage response. Oncogene. 2016;35(2):228–240. 25961923 10.1038/onc.2015.76 PMC4717155

[42] Moretti F . Novel insights about the MDM2/MDM4 heterodimer. Mol Cell Oncol. 2016;3(2):3. 10.1080/23723556.2015.1066923 PMC4905374 27308591

[43] Hajavi J , Ebrahimian M , Sankian M , Khakzad MR , Hashemi M . Optimization of PLGA formulation containing protein or peptide‐based antigen: recent advances. J Biomed Mater Res A. 2018;106(9):2540–2551. 29633511 10.1002/jbm.a.36423

[44] Xia M , Knezevic D , Vassilev LT . p21 does not protect cancer cells from apoptosis induced by nongenotoxic p53 activation. Oncogene. 2011;30(3):346–355. 20871630 10.1038/onc.2010.413

[45] Fischer M , Schwarz R , Riege K , DeCaprio JA , Hoffmann S . TargetGeneReg 2.0: a comprehensive web‐atlas for p53, p63, and cell cycle‐dependent gene regulation. NAR Cancer. 2022;4(1):zcac009. 35350773 10.1093/narcan/zcac009 PMC8946727

[46] Target Gene Regulation Database 2024. Available from: http://www.targetgenereg.org/.

[47] Georgakilas AG , Martin OA , Bonner WM . p21: a two‐faced genome Guardian. Trends Mol Med. 2017;23(4):310–319. 28279624 10.1016/j.molmed.2017.02.001

[48] Goeman F , Strano S , Blandino G . MicroRNAs as key effectors in the p53 network. Int Rev Cell Mol Biol. 2017;333:51–90. 28729028 10.1016/bs.ircmb.2017.04.003

[49] Gembarska A , Luciani F , Fedele C , Russell EA , Dewaele M , Villar S , et al. MDM4 is a key therapeutic target in cutaneous melanoma. Nat Med. 2012;18(8):1239–1247. 22820643 10.1038/nm.2863 PMC3744207

[50] Arena A , Stigliano A , Belcastro E , Giorda E , Rosado MM , Grossi A , et al. p53 activation effect in the balance of T regulatory and effector cell subsets in patients with thyroid cancer and autoimmunity. Front Immunol. 2021;12:728381. 34539667 10.3389/fimmu.2021.728381 PMC8442659

[51] Cortez MA , Ivan C , Valdecanas D , Wang X , Peltier HJ , Ye Y , et al. PDL1 regulation by p53 via miR‐34. J Natl Cancer Inst. 2015;108(1):djv303. 26577528 10.1093/jnci/djv303 PMC4862407

[52] Rinaldo C , Prodosmo A , Mancini F , Iacovelli S , Sacchi A , Moretti F , et al. MDM2‐regulated degradation of HIPK2 prevents p53Ser46 phosphorylation and DNA damage‐induced apoptosis. Mol Cell. 2007;25(5):739–750. 17349959 10.1016/j.molcel.2007.02.008

[53] Wang L , Wang N , Zhang W , Cheng X , Yan Z , Shao G , et al. Therapeutic peptides: current applications and future directions. Signal Transduct Target Ther. 2022;7(1):48. 35165272 10.1038/s41392-022-00904-4 PMC8844085

[54] Michaelis M , Rothweiler F , Barth S , Cinatl J , van Rikxoort M , Löschmann N , et al. Adaptation of cancer cells from different entities to the MDM2 inhibitor nutlin‐3 results in the emergence of p53‐mutated multi‐drug‐resistant cancer cells. Cell Death Dis. 2011;2(12):e243. 22170099 10.1038/cddis.2011.129 PMC3252738

[55] Merlino F , Pecoraro A , Longobardi G , Donati G , Di Leva FS , Brignola C , et al. Development and nanoparticle‐mediated delivery of novel MDM2/MDM4 heterodimer peptide inhibitors to enhance 5‐fluorouracil nucleolar stress in colorectal cancer cells. J Med Chem. 2024;67(3):1812–1824. 38285632 10.1021/acs.jmedchem.3c01312

