# Supplementary material for: Targeting the MDM2‐MDM4 interaction interface reveals an otherwise therapeutically active wild‐type p53 in colorectal cancer
Source: Mol Oncol. 2025 Feb 28;19(8):2412–30. doi: 10.1002/1878-0261.70006 (PMC12330940; doi:10.1002/1878-0261.70006)
Supplement: Supplementary file 1 — Fig. S1. Pep3S reduces clonogenic cell ability. Fig. S2. Pep3S increases cell death of CRC spheroids. Fig. S3. Molecular activities of Pep3S. Fig. S4. Cell cycle analysis. Fig. S5. Pep3S activity in non‐tumor cells. Fig. S6. Effects of p53 and MDM4 on Pep3S activity. Fig. S7. Analysis of PLGA nanoparticles. Table S1. Primers used for qRT‐PCR primers. Table S2. Features of PLGA nanoparticles loaded with Pep3S or Scramble3S. Table S3. Properties of PLGA nanoparticles loaded with vehicle (DMSO), Pep3S, Scramble3S, or FITC‐Pep3S. [file MOL2-19-2412-s001.pdf]

Supplementary Information

Targeting the MDM2-MDM4 interaction interface reveals an otherwise therapeutically active wild-type p53 in colorectal cancer

Sonia Valentini[1,2], Giada Mele[1,2], Marika Attili[1,2], Maria Rita Assenza[1§], Fulvio Saccoccia[1], Francesca Sardina[3], Cinzia Rinaldo[3], Roberto Massari[1], Nicola Tirelli[4], Alfredo Pontecorvi[5], Fabiola Moretti[1]

Table S1: Primers used for qRT-PCR primers

Table S2: Features of PLGA nanoparticles loaded with Pep3S or Scramble3S.

Table S3: Properties of PLGA nanoparticles loaded with vehicle (DMSO), Pep3S, Scramble3S, or FITC-Pep3S.

Fig. S1: Pep3S reduces clonogenic cell ability

Fig. S2: Pep3S increases cell death of CRC spheroids

Fig. S3: Molecular activities of Pep3S

Fig. S4: Cell cycle analysis

Fig. S5: Pep3S activity in non-tumor cells

Fig. S6: Effects of p53 and MDM4 on Pep3S activity

Fig. S7: Analysis of PLGA nanoparticles

Table S1. Primers used for RT-qPCR

Human target gene	Primer sequence 5'-3'
BIK	Fw GACCATGGAGGTTCTTGGCATC<br>Rev CCTGAGGCTCACGTCCATCT
p21	Fw CTGGAGACTCTCAGGGTCGAAA<br>Rev GGCGTTTGGAGTGGTAGAAATC
STAT1	Fw GAAGACCCAATCCAG<br>Rev CCTTGTCCTTCACAT
IGF1R	Fw GCTACGTGAAGATCCGCCATT<br>Rev CTGGTTGTCGAGGACGTAGAAG
SIRT1	Fw GAACCTTTGCCTCATCTGCATT<br>Rev TGGCATATTCACCACCTAACCTAT
SESN2	Fw AGGGACCCGTTGAACAACTCT<br>Rev CCGAGTGAAGTCCTCATATCCGA
MDM4	Fw TCGCACAGGATCACAGTATGG<br>Rev CAGTGTGGGGATATCGTCTTCT
ACTIN	Fw CGCCGCCAGCTCACC<br>Rev CACGATGGAGGGGAA
B2M	Fw CTCGCGCTACTCTCTCTTTCT<br>Rev ACAAAGTCACATGGTTCACACG
RPLP0	Fw ACCTGGAAAACAACCCAGCT<br>Rev GACTCGTTTGTACCCGTTGATG

Table S2. Features of PLGA nanoparticles loaded with Pep3S, or Scramble3S.

Sample	Peptide/PLGA theoretical mass ratio (%)	Peptide/PLGA experimental mass ratio (%)	Z-average (diameter, nm) [1]	PDI [1]	Concentration (particles/mL) [2]	Concentration (Peptide) [3]
PLGA-Pep3S	0.40	0.5	50.5	0.069	2.12e+10 ± 6.50e+08	2.614 µM
PLGA-Scramble3S	0.40	0.6	51.6	0.052	3.36e+10 ± 3.56e+09	2.544 µM

[1] From DLS analysis.

[2] From NTA (Nanosight) analysis.

[3] From fluorescamine assay

Table S3. Properties of PLGA nanoparticles loaded with vehicle (DMSO), Pep3S, Scramble3S, or FITC-Pep3S.

	$\overline{M_w}$ (kDa)[1]	$R_{g,w}$(nm)[2]
PLGA-DMSO	1052 ± 4	13 ± 1
PLGA-Pep3S	428 ± 4	10 ± 1
PLGA-Scramble3S	365 ± 11	13 ± 1
PLGA-FITC-Pep3S	470 ± 2	13 ± 1

[1] $\overline{M_w}$: weight-average nanoparticle mass is calculated from the weight distribution of the nanoparticles obtained via AF4 using SLS and refractive index detection.

[2] $R_{g,w}$: weight-average nanoparticle radius of gyration, obtained as described at point 1 .

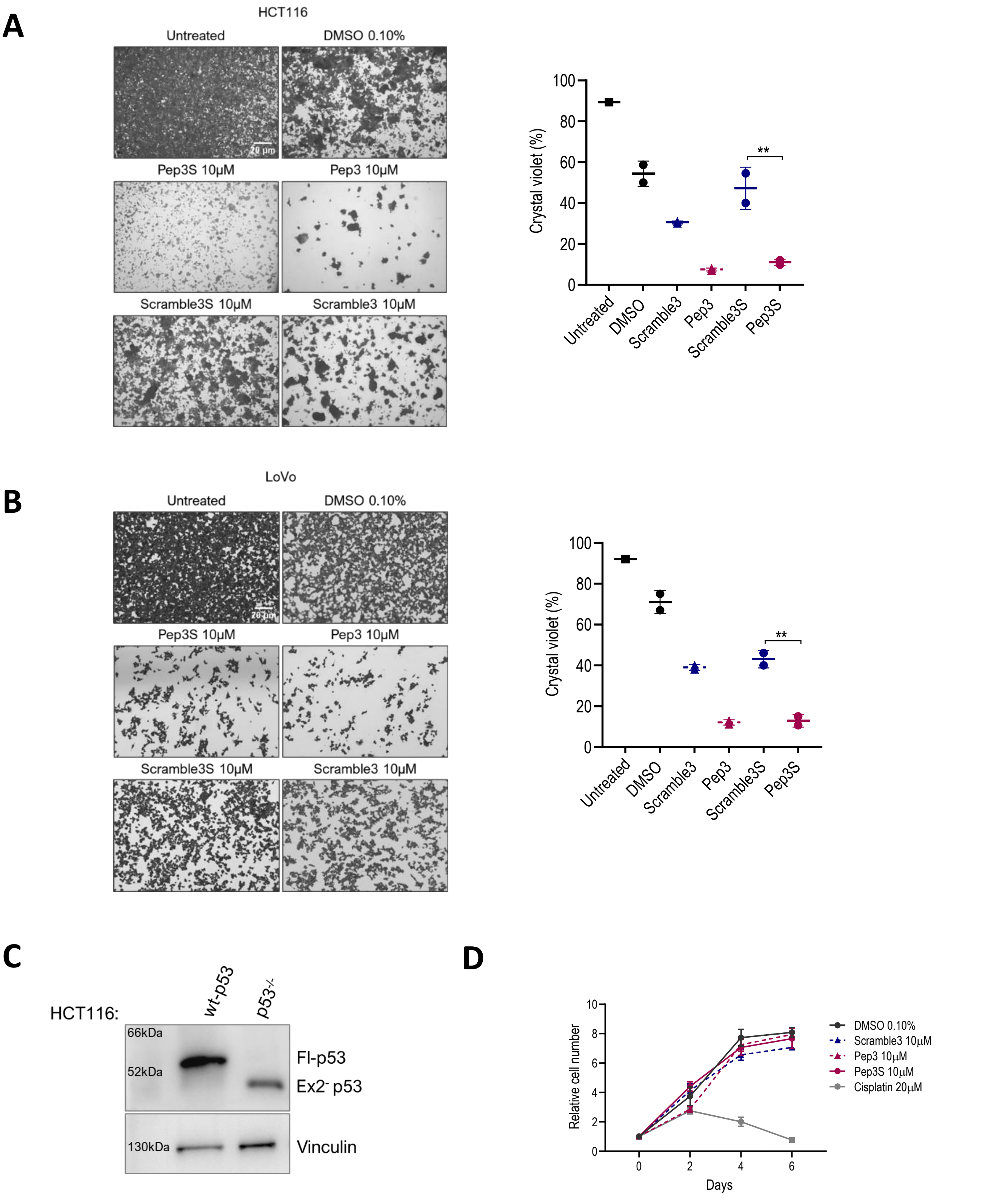

Fig. S1. Pep3S reduces cell clonogenic ability

(A-B), Representative images of long-term cell growth assay after 6 days of treatment of HCT116 (A) and LoVo (B) cells. The percentage of crystal violet was measured through ImageJ. Data on the right is shown as mean ± SD. Scale bar, 20 μm. (C), Western blot analysis of indicated protein of wt-p53 and $p53^{-/-}$ HCT116. (D), Viability assay of $p53^{-/-}$HCT116. Data is from 4 biological replicates. Data is shown as mean ± SD (A, B, D). *P* values were calculated by one-way ANOVA Tukey's multiple comparison test (A, B). **P ≤ 0.01.

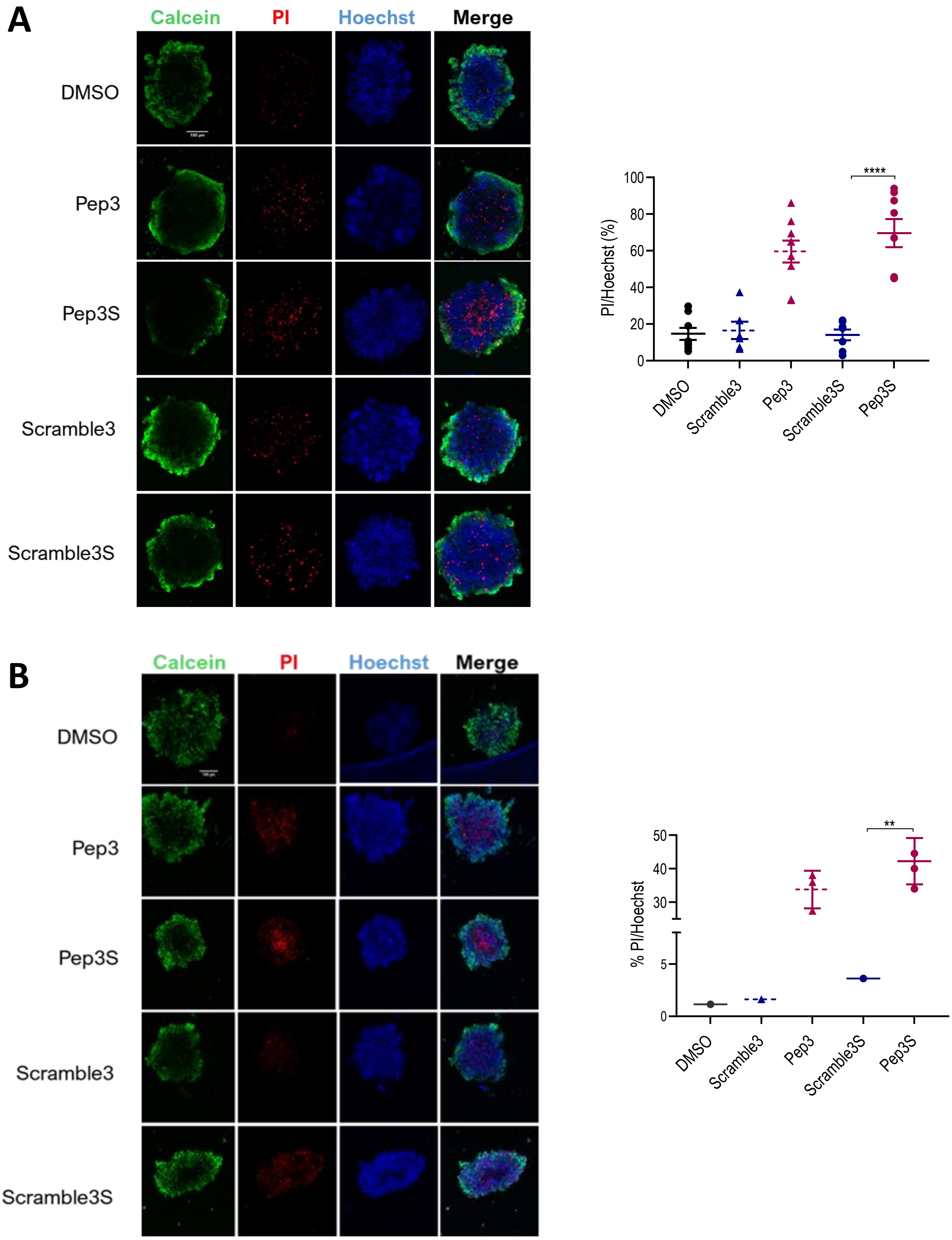

Fig. S2. Pep3S increases cell death of CRC spheroids

(A-B), Representative confocal images of HCT116 (A) and LoVo (B) spheroids after 6 days of treatment. The green signal refers to calcein (live cells), the red to propidium iodide (PI, dead cells), and the blue to Hoechst (nucleic acid stain). Scale bar, 100 µm. The percentage of PI signal was measured through ImageJ and normalized to the Hoechst signal. HCT1116 data is from three independent experiments with two biological replicates and is shown as mean ± SEM. LoVo data is from two independent experiments with two biological replicates and is shown as mean ± SD. P values were calculated by one-way ANOVA Tukey's multiple comparison test. **P ≤ 0.01, ****P ≤ 0.0001.

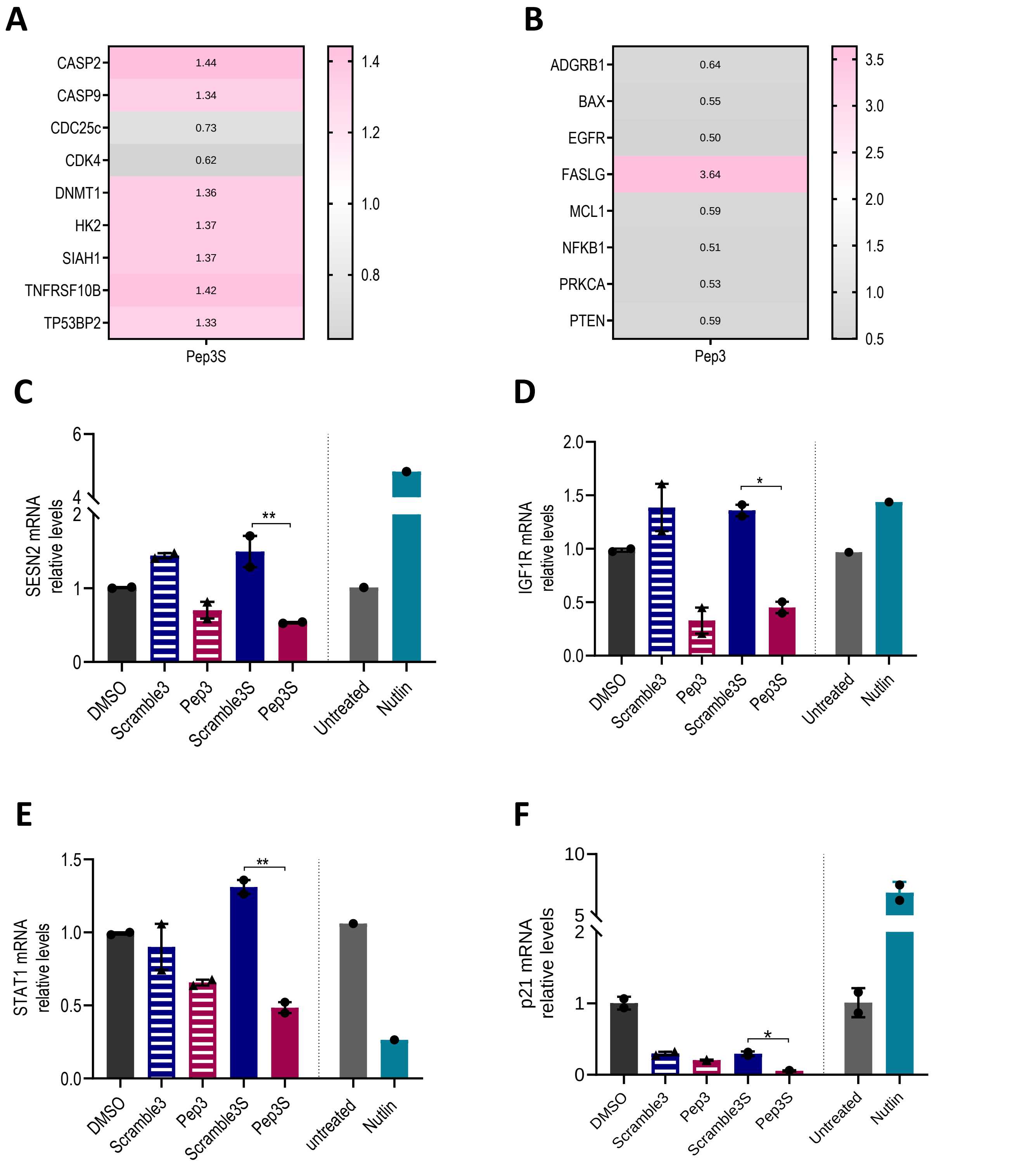

Fig. S3. Molecular activities of Pep3S

(A-B), Heat map of DEGs modulated by Pep3S (A) and Pep3 (B). (C-E), mRNA relative levels by RT-qPCR of SESN2 (C), IGF1R (D) and STAT1 (E) in HCT116 after 48 hrs of treatments. Data is from two independent experiments with two biological replicates. (F), p21 mRNA relative levels by RT-qPCR in LoVo cells after 48 hrs of indicated treatment. Data is from three independent experiments. Data is shown as mean ± SD. P values were calculated by one-way ANOVA Tukey's multiple comparison test (C-F). $^{*}P \leq 0.05$, $^{**}P \leq 0.01$.

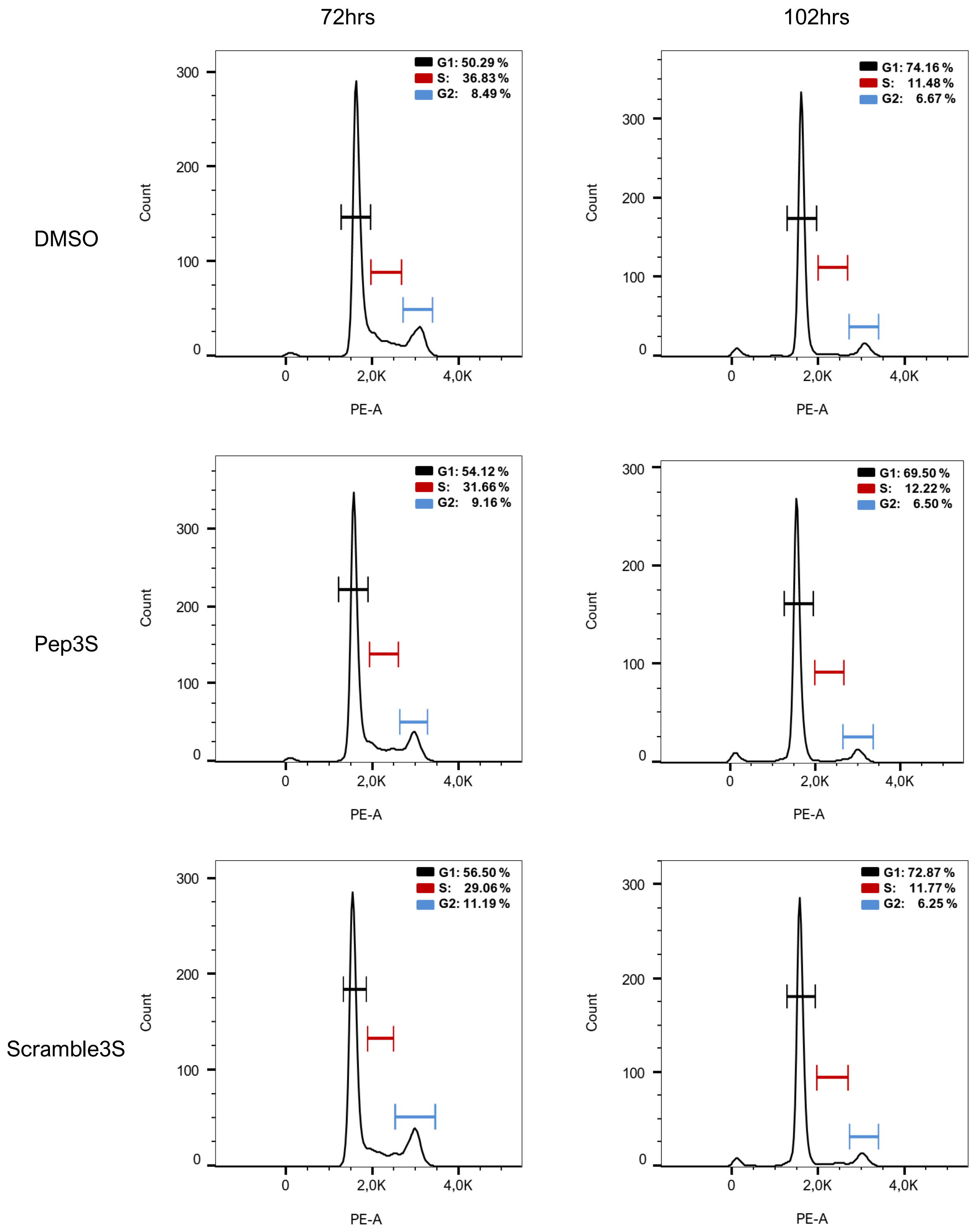

Fig. S4 Cell cycle analysis

Cell cycle histograms of HCT116 treated as indicated for 72 and 102 hrs. The black dash refers to G1, the red to S, and the light blue to G2 phase.

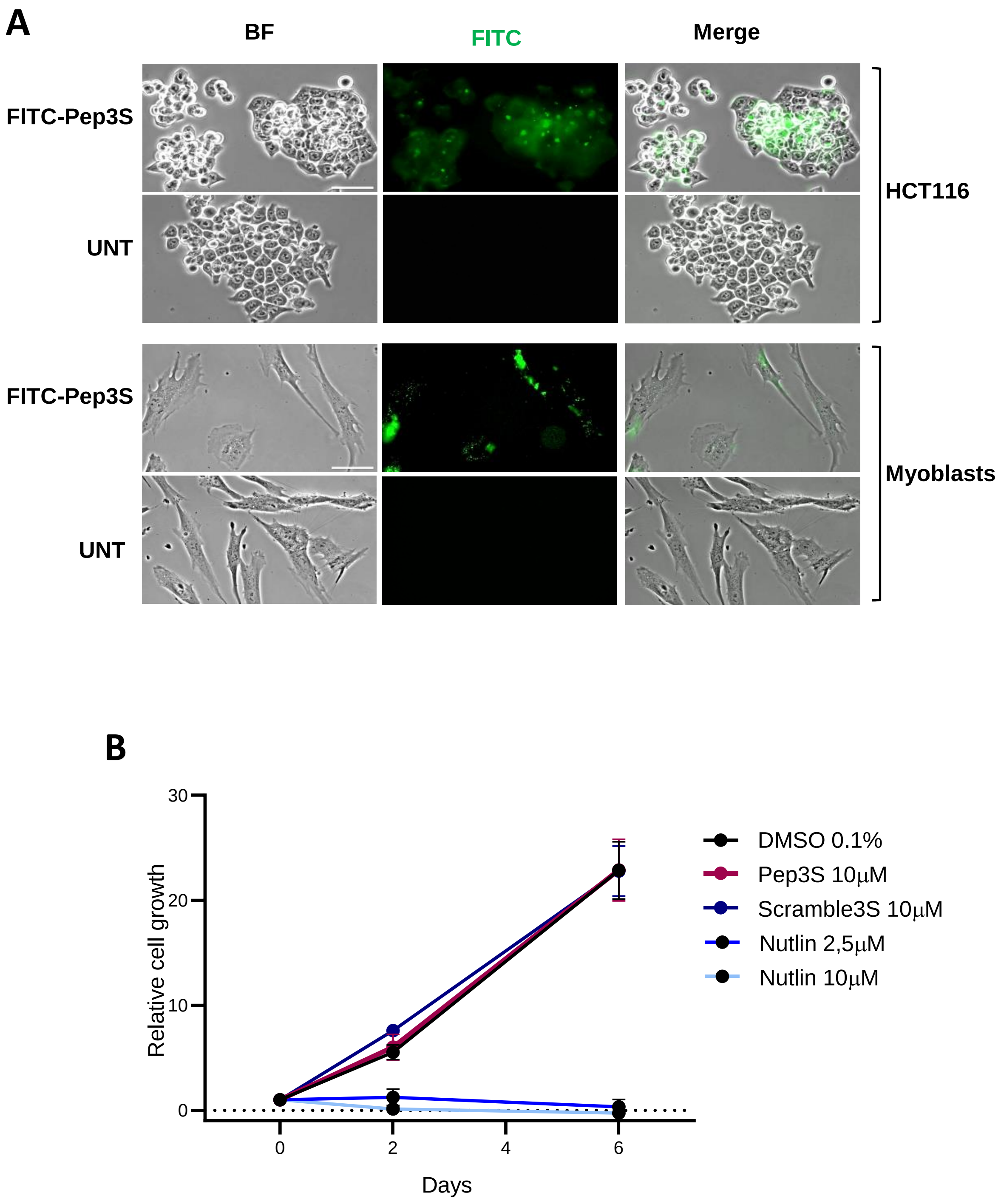

Fig. S5. Pep3S activity in non-tumor cells.
(A), Representative images of HCT116 and myoblasts treated for 24 hours with FITC-Pep3S (10 μM). UNT indicates untreated cells. Scale bar is 50 μm. (B), Viability assay of immortalized lymphoblastoid cells (LCLs) treated as indicated. Data is from 4 biological replicates and is shown as mean ± SD.

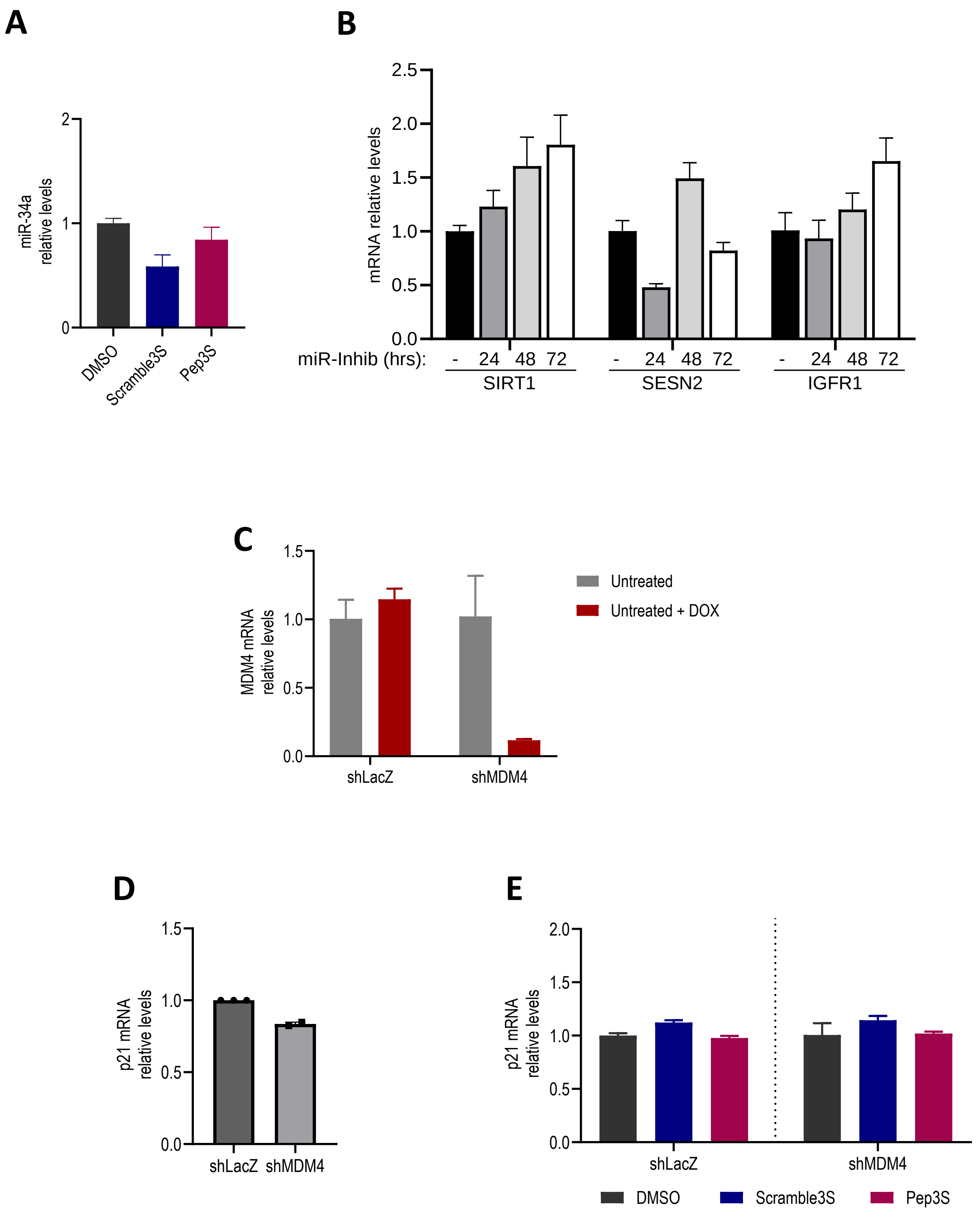

Fig. S6. Effects of p53 and MDM4 on Pep3S activity

(A), miR-34a relative levels by RT-qPCR in $p53^{-/-}$HCT116 cells. (B), mRNA relative levels by RT-qPCR of SIRT1, SESN2, IGF1R at different time points after transient transfection of miR-34a inhibitor in HCT116 cells. All data are relative to the levels of control cells transfected with control siRNA and collected at the same time points of miR-34a inhibitor transfected cells. (C-D), mRNA relative levels by RT-qPCR of MDM4 (C) and p21 (D) in shLacZ and shMDM4 transfected HCT116 after 48 hrs of doxycycline (DOX) treatment. (E), p21 mRNA relative levels by RT-qPCR in shLacZ and shMDM4 transfected $p53^{-/-}$ HCT116 after 48 hrs of indicated treatments. Data is shown as mean ± SD.

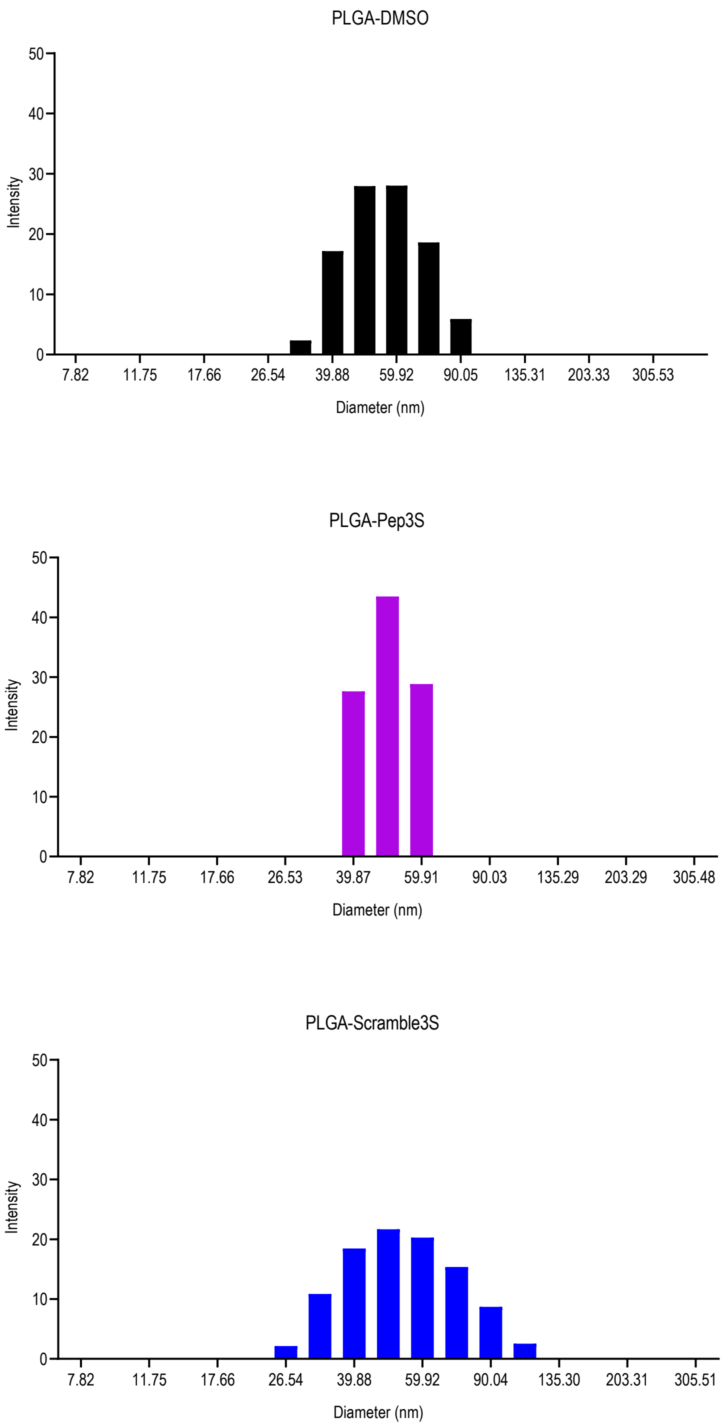

Fig. S7. Analysis of PLGA nanoparticles.

Mean diameter and distribution of PLGA-DMSO (black), PLGA-Pep3S (violet), and PLGA-Scramble3S (blue) by dynamic light scattering (DLS) after filtration.
